# Differential fates of Kazald gene quartet: Ancestral roles in skeletogenesis and regeneration to putative innovations in fish and birds

**DOI:** 10.1016/j.isci.2026.114934

**Published:** 2026-02-10

**Authors:** Sean D. Keeley, Rita Aires, Belfran Carbonell Medina, Claudia Marcela Arenas-Gómez, Alejandra Cristina López-Delgado, Jean Paul Delgado, Franziska Knopf, Shigehiro Kuraku, Tatiana Sandoval-Guzmán

**Affiliations:** 1 Department of Internal Medicine III, University Hospital Carl Gustav Carus, Technische Universität Dresden, 01307 Dresden, Germany; 2 Center for Healthy Aging, University Hospital Carl Gustav Carus, Technische Universität Dresden, 01307 Dresden, Germany; 3 Center for Regenerative Therapies, Technische Universität Dresden, 01307 Dresden, Germany; 4 Grupo de Génetica, Regeneración y Cáncer – Departamento de Estudios Básicos Integrados, Facultad de Odontología, Universidad de Antioquia UdeA, Medellín 050010, Colombia; 5 Grupo de Biodiversidad para la Sociedad, Dirección Académica, Universidad Nacional de Colombia, Sede de La Paz, La Paz 202017, Colombia; 6 Grupo Genética, Regeneración y Cáncer, Facultad de Ciencias Exactas y Naturales, Instituto de Biología, Universidad de Antioquia UdeA, Medellín 050010, Colombia; 7 Molecular Life History Laboratory, Department of Genomics and Evolutionary Biology, National Institute of Genetics, Mishima 411-8540, Japan; 8 Department of Genetics, Graduate University for Advanced Studies, SOKENDAI, Mishima 411-8540, Japan; 9 Paul Langerhans Institute Dresden, Helmholtz Centre Munich, University Hospital Carl Gustav Carus, Technische Universität Dresden, 01307 Dresden, Germany

**Keywords:** Natural sciences, Evolutionary biology, Evolutionary developmental biology, Evolutionary history

## Abstract

Gene orthology inference across species is crucial to identify gains and losses of functions. The discovery in axolotl (*Ambystoma mexicanum*) of a regeneration-associated gene identified as either *Kazald1* or *Kazald2* led us to investigate its evolution via an extensive cross-species analysis. Molecular phylogeny inference identified the gene as *Kazald2* and revealed an undescribed four-member Kazald gene family in jawed vertebrates. Synteny comparisons demonstrated that this family originated in the two-round whole-genome duplication event. Additionally, vertebrate-wide comparisons of Kazald expression, validated in tissues of axolotl, zebrafish, and sharks, uncovered seemingly ancestral connections conserved over jawed vertebrate evolution, and suggested novel putative roles within specific lineages. Our study demonstrates the establishment of the Kazald family in the jawed vertebrate ancestor and elucidates the asymmetry of gene fates of its members. This provides a comprehensive report of this formerly undescribed gene family, offering a solid foundation for its study in diverse species.

## Highlights

• Kazald gene family originated in Bilateria, with 4 paralogs in jawed vertebrates
• *Kazald1* and *Kazald2* have conserved expression in bone and regeneration respectively
• *Kazald3* is expressed instead in the skeleton and during regeneration in teleosts
• Birds have evolved a unique expression profile of *Kazald4* within their brains

## Graphical abstract

**Figure:**
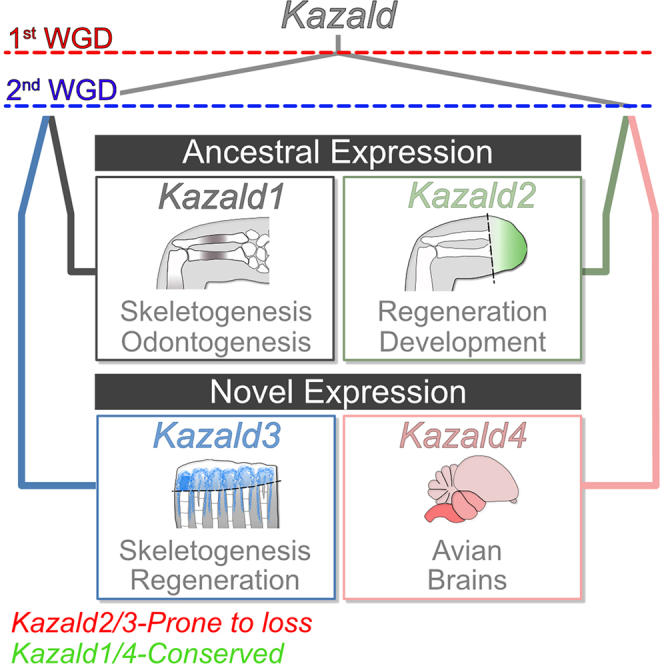

## Introduction

Regenerative ability greatly differs across the animal Tree of Life, from lineages with limited capacity, such as mammals and birds, to those such as salamanders and teleost fish, that can regenerate complex structures, and all the way to species capable of whole-body regeneration such as hydra, planaria, and sea stars.^1^^,^^2^^,^^3^ This discrepancy in ability has driven research into how this process varies across species to uncover its underlying mechanisms. A common idea is that the ability to regenerate diverse tissues is ancestral to vertebrates but has been lost or suppressed in many tetrapod lineages.^4^ Therefore, the pathways used for regeneration in certain species may still be present in regeneratively limited ones, such as mammals, but are now utilized for different purposes and no longer activated by injury.

Several genes have been identified as promising candidates for reactivating these pathways and inducing successful regeneration.^5^^,^^6^^,^^7^^,^^8^^,^^9^ One candidate gene was found in the axolotl salamander (*Ambystoma mexicanum*), and originally identified as *Kazal-type serine peptidase inhibitor domain 1* (*Kazald1*). This gene is heavily expressed in the blastema of regenerating limbs but not in developing limb buds, and its knockdown significantly slowed and reduced the regenerative response.^5^ This resulted in widespread interest in this gene, as it appeared both specific to, and strongly impactful on, regeneration. However, subsequent studies in other species, such as *Xenopus* frogs and *Acomys* spiny mice, did not find similar upregulation of their *Kazald1* genes in regenerating tissues.^10^^,^^11^ Furthermore, different studies and reviews on the axolotl have been inconsistent in referring to this gene, sometimes labeling it as *Kazald2* instead of *Kazald1*.^7^^,^^12^^,^^13^

*Kazald2* is originally known from zebrafish, and outside the potential presence in axolotl, has not been found in examined tetrapods.^14^ *Kazald2* is also absent from several other species of fish, including the pufferfish (*Takifugu rubripes*) and stickleback (*Gasterosteus aculeatus*).^14^ Rather, these species only possess a related gene, *Kazald3*, which is also present in zebrafish and, similar to *Kazald2*, was not found in any analyzed tetrapod. Instead, all investigated tetrapods maintained only a singular Kazald gene, *Kazald1*, which was not identified in any examined teleost fish. While not labeled as a Kazald gene, *Mixer inducible gene 30* (*Mig30*) in *Xenopus laevis* was found to be related to *Kazald1* (then named *IGFBP-rP10*).^15^ However, the relationship of *Mig30* to the teleost *Kazald2* or *Kazald3* has never been investigated. Thus, if the regeneration-associated axolotl gene and/or the *X. laevis Mig30* were orthologous to either of the teleost Kazald genes, it would reveal that those genes were not lost in the tetrapod ancestor, as the available data have previously suggested.

Correct identification of the axolotl gene is also of particular concern since inaccurate gene naming can create long-standing problems. Instances of confusion due to the maintenance of different paralogs between species, known as “hidden paralogy,”^16^ can result in orthologous genes being assigned different names across them, as was once the case with amniote vs. zebrafish *Wnt11*.^17^ This can greatly complicate investigations of genes within published research or online databases, and thus, previous discoveries are prone to being overlooked. Alternatively, non-orthologous genes may be given the same name, as has occurred in mammal vs. chicken Nodal gene families.^17^ When this occurs, discoveries made about a gene in one species may incorrectly be attributed to the non-orthologous gene of the other species. Additionally, any differences between the two genes will erroneously be thought to be evolutionary adaptations of those lineages.

Finally, updating gene names to match their orthology can be very difficult, especially if the field grows accustomed to the inaccurate nomenclature, resulting in the incorrect and/or conflicting terminology being used for a long time.^18^ Therefore, it is important to correctly identify genes as early as possible to avoid greater confusion later on. While a previous study of ours made use of online phylogenetic tools to support the choice of *Kazald2* for this axolotl gene,^12^ a more focused and exhaustive phylogenetic analysis was still needed to definitively classify its identity and relationship to the Kazald genes of other species. This would enable the more accurate determination of when any roles associated with these genes arose.

Currently, most knowledge is centered on *Kazald1* in mammals, where it promotes the proliferation of osteoblastic cells during skeletogenesis and is expressed during odontogenesis.^19^^,^^20^ *Kazald1* also induces the growth and invasion of cancer cell lines, and its hypomethylation was associated with accelerated cancer progression.^21^^,^^22^ This influence on proliferation may also extend beyond mammals, as it is expressed during embryogenesis in *X. laevis*.^15^ However, much less is known about the roles of *Kazald2*, *Kazald3*, and *Mig30*. In zebrafish, *Kazald2* is transcribed during development in the epiblast at the dome and shield stages, and in a subpopulation of cranial-pharyngeal mesoderm at 13 hours postfertilization.^23^^,^^24^ Meanwhile, no expression profile has been identified for *Kazald3* to our knowledge. Finally, *Mig30* is expressed in multiple embryonic tissues in *X. laevis* and impacts head formation and morphogenetic movements during gastrulation.^15^^,^^25^

To characterize the relationships of these genes and uncover conserved and novel expression profiles across organisms, we identified putative Kazald genes in approximately 60 vertebrate species spanning the Tree of Life, as well as several invertebrate deuterostome and protostome species representing major lineages within them. Molecular phylogeny analysis conclusively demonstrated that the regeneration-associated axolotl gene is *Kazald2*. Even more importantly, it discovered it was part of a larger Kazald gene family that originated in the bilaterian ancestor, and which consists of four paralogs in jawed vertebrates. Synteny analysis uncovered that these paralogs arose in the two-round whole-genome duplication (2R-WGD) event ancestral to jawed vertebrates. Additionally, to our knowledge, one of these paralogs has never been formally described, and thus we identify it here as *Kazald4*. We also provide a thorough account of which lineages still maintain specific Kazald genes, and by analyzing RNA-Seq expression data and performing RNA *in situ* hybridization and RT-qPCR, we have found several tissues, including the brain and bones, and biological processes, such as regeneration and development, in which these genes are expressed across species. This will provide the research community with a fundamental knowledge of these genes, which would otherwise often be overlooked due to the large difficulties incurred by a lack of annotation and prior existing information. In this way, this work will also act as a solid foundation for future studies focused on individual Kazald genes and the mechanisms through which they work.

## Results

### Identification of Kazald genes in the axolotl

To determine if the regeneration-associated axolotl Kazald gene was orthologous to *Kazald1*, *Kazald2*, or a salamander-unique gene, the axolotl transcriptome and genome were searched via Reciprocal Best Hit (RBH) BLAST^26^ (Table 1; Table S1). For *Kazald1*, the mouse gene was our representative sequence due to it being supported by many publications.^19^^,^^20^ For *Kazald2*, we utilized the zebrafish gene as it was the only major model organism known to possess it, and experimental evidence supports the accuracy of its sequence. We also performed a search with zebrafish *kazald3* since it was the only other gene expressly identified as a Kazald gene. This discovered four distinct genes, spread across four different chromosomes. Additionally, the regeneration-associated axolotl gene (XM_069632371.1) was not the most similar one to mouse *Kazald1*, strengthening the hypothesis that it is not the *Kazald1* ortholog. However, its comparably low similarity to zebrafish *kazald2* prevented any obvious conclusions about potential orthology between these two genes. Furthermore, there being more Kazald genes in the axolotl than in the mouse and zebrafish combined, greatly complicated identifying evolutionary relationships within these species. Thus, we expanded our examination to include diverse species across the animal Tree of Life.

**Table 1:** TBLASTN search reveals the presence of four axolotl genes with a high similarity to known Kazald genes

Axolotl Gene	Identities	Positives	E Value	Chromosome
**Mouse *Kazald1***				

XM_069611649.1	179/249 (72%)	207/249 (83%)	9e-128	Chr8q
XM_069654333.1	133/243 (55%)	162/243 (67%)	3e-88	Chr3q
XM_069617721.1	118/225 (52%)	152/225 (68%)	2e-70	Chr10q
**XM_069632371.1**	112/233 (48%)	151/233 (65%)	3e-66	Chr6q

**Zebrafish *kazald2***				

**XM_069632371.1**	132/270 (49%)	168/270 (62%)	8e-74	Chr6q
XM_069654333.1	117/274 (43%)	163/274 (59%)	2e-68	Chr3q
XM_069611649.1	106/234 (45%)	143/234 (61%)	3e-60	Chr8q
XM_069617721.1	108/228 (47%)	140/228 (61%)	3e-58	Chr10q

Identities are the number of identical amino acids. Positives are aligned amino acids that are either identical or have similar chemical properties. E Value is the number of expected hits of similar quality that could be found just by chance. Chromosome indicates where the axolotl gene is located. Axolotl gene IDs taken from UKY_AmexF1_1 genome assembly (GCF_040938575.1). Regeneration-associated axolotl gene is bolded.

### The jawed vertebrate ancestor possessed four unique Kazald genes

Our examination began within jawed vertebrates (gnathostomes) via a TBLASTN search utilizing the seven aforementioned Kazald genes. This included 61 species from its three major lineages: the sarcopterygians, including 38 tetrapod species from both amniote and amphibian clades, a lungfish, and a coelacanth; the actinopterygians, comprising 14 teleost and 4 non-teleost species; and the chondrichthyans, consisting of a shark, a ray, and a chimaera. Outside the jawed vertebrates, two representative jawless fish species (cyclostomes) and several different invertebrates were included (Table S2). The latter encompassed deuterostomes, including chordates and echinoderms; protostomes, such as arthropods, mollusks, and annelids; and several non-bilaterians. This found that Kazald genes are present throughout deuterostomes and protostomes, but not in cnidarians, placozoans, or sponges (Table S2). Furthermore, we did not even find genes in non-bilaterians that possessed all three protein domains of *Kazald1*, i.e., IGFBP, Kazal, and IG-like,^19^ although cnidarians did possess some genes with the IGFBP and Kazal domains in close proximity. Thus, the ancestral Kazald gene appears to have originated in bilaterians. Additionally, our search through bilaterian invertebrates uncovered that their RBH matches to Kazald genes were often instead identified as *insulin-like growth factor binding protein-related protein 1* (*IGFBP-rP1*; i.e., *Igfbp7*). However, except for in amphioxi and the tunicate *Clavelina lepadiformis*, we did not identify any invertebrates possessing genes with RBH matches to mouse or axolotl *Igfbp7*. Thus, we suggest that *Igfbp7* is a unique chordate development, and that instances of *IGFBP-rP1*/*Igfbp7* genes in non-chordates are, in actuality, Kazald genes.

This analysis also revealed that most vertebrate lineages possessed multiple Kazald genes, with notable exceptions being cyclostomes, snakes, and placental mammals, which only had one (Table S2). In contrast, most invertebrates only possessed one Kazald gene, except for some species from lineages known to have experienced whole-genome duplications, such as horseshoe crabs.^27^ This raised the idea that gnathostome Kazald genes were also established via the two-round whole-genome duplication event ancestral to this lineage.^28^^,^^29^ However, the lack of multiple Kazald genes in cyclostomes, which also experienced multiple genome duplications,^30^^,^^31^ and no chondrichthyans having more than two Kazald genes, left open the possibility that at least some of these genes were unique developments within bony vertebrates (osteichthyans). Thus, we performed a comprehensive phylogenetic analysis via maximum likelihood (ML) and Bayesian inference (BI) using the amino acid sequences of all our identified Kazald genes.

**Figure 1:**
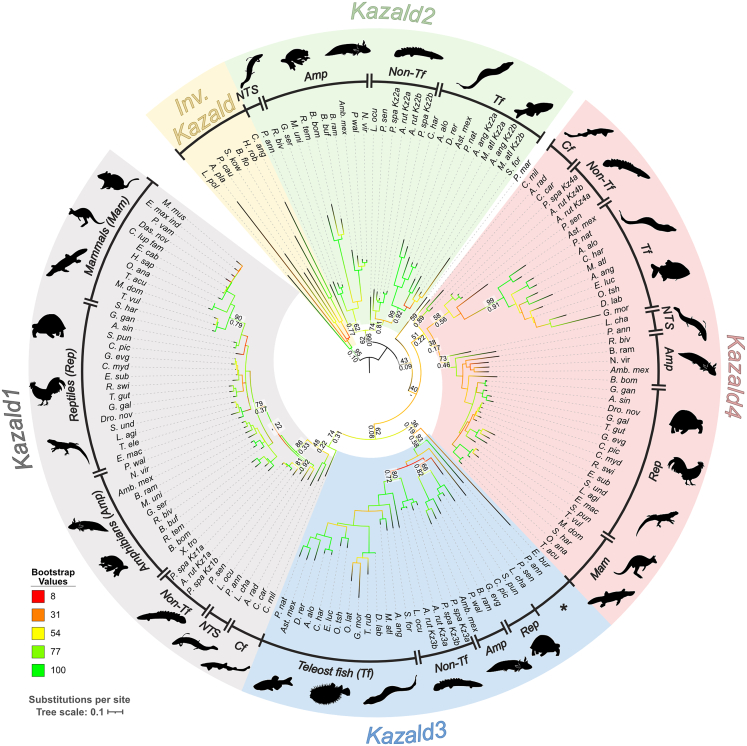
Phylogenetic tree of Kazald genes reveals that all vertebrate genes are split into four distinct clades Displayed consensus tree was generated via RAxML using amino acid sequences. Branch colors reflect the bootstrap values of each node, and do not correspond to branch support. Support values are shown at several key evolutionary nodes, e.g., at the base of mammals, amniotes, and teleost fish, or when the values between maximum likelihood (ML) and Bayesian inference (BI) trees differ considerably. Layout of the support values is: ML bootstrap support via RAxML (top), BI posterior probabilities via BAli-Phy (bottom). Species are grouped into major categories within each Kazald gene clade. Inv. = invertebrates, NTS = non-tetrapod sarcopterygians, Cf. = cartilaginous fish, Non-Tf = non-teleost ray-finned fish. ∗ marks a polyphyletic grouping containing a jawless vertebrate, two non-tetrapod sarcopterygians, and one non-teleost ray-finned fish. Abbreviated species names are listed in Table S2 and Data S1. Kazald a and b versions created by whole-genome duplications that occurred after the 2R-WGD event in certain fish lineages are marked next to the abbreviated species name. Scale bar corresponds to the mean number of amino acid substitutions per site.

This discovered that all jawed vertebrate Kazald genes were part of a single gene family, comprising four distinct clades (Figure 1). This was supported by both phylogenetic methods, with their generated trees being extremely similar despite low posterior probabilities (PPs) at basal nodes in the BI tree (e.g., 0.10 PP vs. 95 Bootstrap for the invertebrate Kazald node, and 0.08 PP vs. 62 Bootstrap for the clade grouping *Kazald1* with *Kazald3*). As BAli-Phy is notoriously slow when handling large quantities of sequences,^32^ a potential explanation for these low values is that convergence at these basal nodes was not reached when analysis was terminated. This could be especially possible if genes with low placement support, such as those of the lamprey and hagfish, varied in position across the set of trees due to ambiguity or conflict in their phylogenetic signal, making them act as rogue taxa.^33^^,^^34^ As removal of rogue taxa can greatly increase resolution and branch support values within the consensus tree,^35^^,^^36^ phylogenetic analyses were rerun using a subset of Kazald genes. This resulted in even higher support in both ML and BI trees for the four jawed vertebrate Kazald clades predicted by our initial large-scale analysis (Figure S1). Phylogeny inference using PhyloBayes with the CAT-GTR model was also performed to try to increase confidence in the cyclostome Kazald gene relationships, as it is more robust to long branch attraction.^37^ This closely matched the initial trees, but with increased support for the separation of the hagfish and lamprey Kazald genes (Figure S2).

Three of these clades contained at least one previously validated gene from a major model species: *Kazald1* of human and mouse, and *kazald2* and *kazald3* of zebrafish. Therefore, the clades containing these genes were assigned the corresponding ID. Finally, to maintain consistency with the established sequential naming convention, we classified the remaining unlabeled clade as *Kazald4*, which, to our knowledge, has never been previously described. Importantly, the two chondrichthyan Kazald genes are *Kazald1* and *Kazald4*. As *Kazald2* is the outgroup to these genes but is possessed by several osteichthyan lineages, it was most likely present in the jawed vertebrate ancestor and later lost in chondrichthyans. Furthermore, the Kazald genes of lamprey and hagfish, representing the two major cyclostome lineages, were also part of different Kazald clades, potentially indicating that these genes are not orthologous and existed prior to the cyclostome-gnathostome split, although their low support values prevented making such a conclusion without additional data.

### Kazald gene quartet was established via two rounds of whole-genome duplication

The presence of four distinct Kazald gene clades within the gnathostome lineage, with at least three most likely being present prior to the osteichthyan-chondrichthyan split, further supported their generation from a singular invertebrate Kazald gene ancestor via the 2R-WGD event. If this were the case, then the surrounding genes would have also been replicated. Furthermore, phylogenetic relationships between the resulting paralogs of each of these surrounding genes should be similar to that of the Kazald genes. Thus, we checked for conserved intragenomic synteny, i.e., the conservation of a similar array of paralogous genes,^38^^,^^39^ between the genomic regions containing Kazald paralogs within a species.

**Figure 2:**
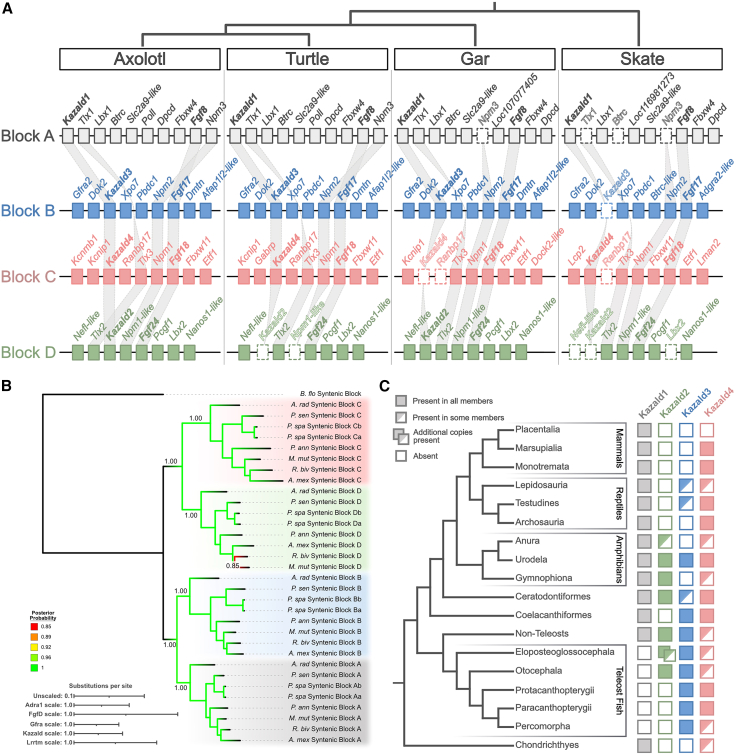
Conserved intra-/intergenomic synteny analysis supports the origin of the Kazald gene family in the 2R-WGD event ancestral to jawed vertebrates (A) Illustration of the syntenic blocks containing the Kazald genes in four representative species. Various paralogous genes are connected via gray lines. Genes lost in a species are drawn with a dotted outline and no internal color. Kazald genes are bolded. Fgf genes were used as the syntenic block anchor when a Kazald gene was absent, and thus are also bolded. (B) Consensus tree generated via BAli-Phy partitioned analysis using the amino acid sequences of the five listed gene families (see also Figure S3). Branch colors indicate the BI posterior probability at each node. Support values shown at nodes are BI posterior probabilities. Scale bar corresponds to the mean number of amino acid substitutions per site for each of the used gene families. (C) Simplified diagram of individual Kazald gene maintenance across vertebrates, along with the relationships of the different lineages. Colors of Kazald genes and the syntenic blocks correspond to each other. Dual boxes for *Kazald2* in Eloposteoglossocephala represent the presence of *Kazald2a* and *Kazald2b* in the contained superorder Elopomorpha. Urodela is listed with the four Kazald genes present, as every examined species with a sequenced genome was found to possess all four genes.

Species were selected that each contained as many of the four Kazald genes as possible, while also representing sarcopterygians, actinopterygians, and chondrichthyans. Teleost fish were excluded due to complications caused by their additional whole-genome duplication. Ultimately, a species of salamander (*Ambystoma mexicanum*), turtle (*Mauremys mutica*), lungfish (*Protopterus annectens*), bichir (*Polypterus senegalus*), gar (*Lepisosteus oculatus*), and skate (*Amblyraja radiata*) were used, with four large genomic regions identified in each species. These were categorized as syntenic blocks A – D, based on their contained Kazald and fibroblast growth factor subfamily D (FgfD)^40^ gene. Regions of these blocks immediately surrounding the Kazald genes in a subset of these species are illustrated in Figure 2A.

This confirmed the existence of intragenomic synteny, with several families of known paralogous genes distributed across these syntenic blocks in each species. Furthermore, some sets of paralogous genes, such as *Fgf8*/*17*/*18*/*24* and *T cell leukemia homeobox 1/2/3* (*Tlx1*/*2*/*3*), have previously been hypothesized to originate in the 2R-WGD event.^14^^,^^41^^,^^42^ Finally, the large-scale regions of the human and gar genomes containing these syntenic blocks roughly correspond to the locations encompassing Chordate Linkage Group I (CLGI) and Group Q (CLGQ),^43^ further bolstering the idea that these regions are the products of the 2R-WGD event. Subsequent examination of the sea lamprey (*Petromyzon marinus*) and brown hagfish (*Eptatretus atami*) genomes found their Kazald genes located on chromosomes 9 and 2, respectively, indicating that CLGI specifically was the linkage group that contained the ancestral vertebrate Kazald gene.^30^ Moreover, these cyclostome chromosomes not being orthologous to each other^30^ revealed that the lamprey and hagfish Kazald genes are not orthologs, which was suggested by our molecular phylogeny inference but remained uncertain due to the low bootstrap and posterior probability support values.

Apart from conserved intragenomic synteny within individual species, we observed conserved intergenomic synteny of the syntenic blocks across species. For example, the gene identified as *Kazald1* in a species was always associated with specific members of the aforementioned gene families, such as *ladybird homeobox 1* (*Lbx1*) and *Fgf8*, as well as with genes that lacked paralogs elsewhere in the genome, such as *deleted in primary ciliary dyskinesia homolog (mouse)* (*Dpcd*). This conserved intergenomic synteny greatly supports there being exactly four Kazald clades in jawed vertebrates, as predicted in our initial tree (Figure 1), and that genes within individual species were attributed to the appropriate clade. It also highlights other interesting evolutionary findings, such as that *Fgf24*, which was previously thought to be lost in tetrapods,^44^^,^^45^ is actually still present in axolotl and turtles (Figure 2A).

Finally, we analyzed the phylogenetic relationships of five gene families – adrenoceptor alpha 1 (Adra1), FgfD, GDNF family receptor alpha (Gfra), Kazald, and leucine rich repeat transmembrane neuronal (Lrrtm) – that still had a paralog present within each syntenic block in the majority of our selected species. While the exact structure of the gene trees sometimes differed, there was no discordance of which syntenic blocks were most closely related, e.g., syntenic block C was never more closely related to syntenic block A than it was to syntenic block D (Figure S3). Additionally, no genes from a particular syntenic block were split apart, or mixed with genes of another syntenic block.

Thus, with no conflict between individual gene trees, we conducted a partitioned analysis using all of these gene families. This approach can more accurately reflect true evolutionary histories by uncovering hidden support for internal relationships otherwise lost when only using individual gene families.^46^^,^^47^ Additionally, using multiple genes acts as a better proxy for the complete syntenic blocks, and thus more accurately reflects the evolution of these genomic regions. Our partitioned multigene tree maintained the pairing of *Kazald1* (syntenic block A) with *Kazald3* (syntenic block B), now with even higher confidence (Figure 2B). However, *Kazald4* (syntenic block C) and *Kazald2* (syntenic block D) are now grouped together with very high confidence, matching what would be expected of four genomic regions originating from two sequential genome duplications. These relationships between syntenic blocks and the chromosomes on which these blocks reside in the gar and chicken, enabled us to associate syntenic blocks A/B/C/D with the CLGI copies 1α/1β/2β/2α, respectively. Thus, we can conclude that the Kazald gene family was created through the 2R-WGD event, and therefore all four genes were present in the gnathostome ancestor.

### Kazald4 is highly expressed in avian brains

With the phylogeny of the syntenic blocks predicted with high confidence, an overview of Kazald gene maintenance across vertebrates could be achieved, revealing that it differed greatly across lineages (Figure 2C). However, some were more likely to be maintained than others, with *Kazald1* and *Kazald4* being the first and second-most consistently conserved genes across tetrapods, respectively. Surprisingly, despite *Kazald4* being present in salamanders, lizards, birds, and even non-placental mammals, we could not find a formal report of it or its expression. This was unexpected for such a widely conserved gene, and so we examined public gene expression atlases for potential expression profiles.^48^

**Figure 3:**
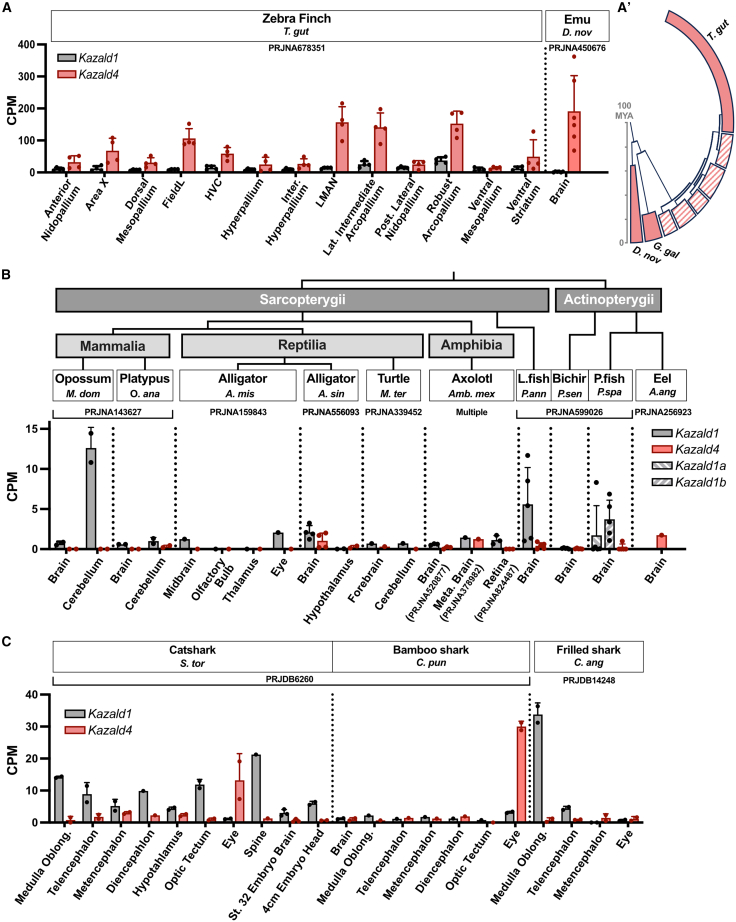
*Kazald4* is highly expressed in avian brains, but not in the brains of other species (A) Quantification of Kazald gene expression in different parts of the zebra finch brain and in the whole emu brain. (A′) shows a simplified evolutionary tree of birds (based on^49^); clades containing species shown to express *Kazald4* in the brain are filled in, while remaining clades have a striped pattern. (B) Quantification of *Kazald1* and *Kazald4* gene expression in the brains and retinas of non-avian osteichthyans. A simplified overview of the evolutionary relationships of the examined species is displayed above the gene expression graph. *Kazald1* was duplicated in the lineage leading to paddlefish (P.fish) with no subsequent loss, creating *Kazald1a* and *Kazald1b*. L.fish = Lungfish. (C) Quantification of Kazald gene expression in different parts of the brain and associated neural tissues of several chondrichthyans. PRJ IDs indicate the publicly available RNA-Seq datasets that generated the raw data for the listed tissues. Dots represent biological replicates in examined datasets, and error bars represent standard deviation when calculable. CPM = counts per million. Data are represented as Mean ± SD.

This found that *KAZALD4* (current annotation: LOC769726) is highly expressed in many parts of chicken brain. Analyzing published RNA-Seq data of other avian species found that *Kazald4* was similarly highly expressed in the whole brain of emu and various parts of the brain of zebra finch (Figure 3A). As these species span the entire avian lineage, *Kazald4* is most likely expressed in the brains of birds in general (Figure 3A′). However, *Kazald4* was not similarly expressed in data generated from brains of other bony vertebrates (Figure 3B). This included alligators of the order Crocodilia, the most closely related extant lineage to birds, indicating that its expression in avian brains is likely a unique adaptation in this lineage. However, we could not disregard the possibility that this adaptation arose earlier within the archosaur lineage, but was then lost in the branch leading to Crocodilia.

Interestingly, data from some shark species demonstrated *Kazald4* expression in eyes, a region of which constitutes part of the central nervous system^50^ (Figure 3C). However, it could not be determined if *Kazald4* was specifically expressed in the neural tissue of the eye. Since our genetic and proteomic analysis of the four Kazald genes found strong similarities in their exon-intron structure, layout of protein domains, and the majority of their 3D protein structure (Figures S4 and S5), we investigated if a different Kazald gene could be standing in for it, thus explaining the lack of *Kazald4* expression in non-avian brains. This revealed noticeable levels of *Kazald1* in at least parts of the brain of a few jawed vertebrates, including some sharks, lungfish, opossum, and zebra finch (Figures 3A–3C). However, most species did not express any Kazald gene in their brains (Figure S6). Additionally, there was little to no Kazald gene expression in the brain or nerves in non-gnathostome deuterostomes, such as hagfish, lampreys, amphioxi, and tunicates, with the exception of some low-to-moderate presence in nerves of the starfish *Acanthaster planci* (Figures S7D–S7F). Thus, it appears that, except for birds, there is no strong connection of any Kazald gene to the brain in jawed vertebrates that is consistent within a wider lineage, and that a Kazald gene becoming expressed in neural tissue is also rather uncommon within deuterostomes.

Exploring outside of the brain, we also did not observe much, if any, *Kazald4* expression across most species in other tissues, nor during major biological processes such as development or regeneration (Figures S8C and S8D). While some individual species of lungfish and sharks did express *Kazald4* in various tissues, these expression profiles were not recapitulated in other species within their lineage or closely related ones (Figures S8A and S8B). We additionally did not find a tissue that consistently expressed a Kazald gene across cyclostomes (Figure S7D). While part of this discrepancy could be due to RNA-Seq data coming from multiple sources, generated via diverse experimental approaches with different resolutions, some of these differences between species were observed even within the same dataset. One notable example is seen in the differences in *Kazald4* expression between the catshark *Scyliorhinus torazame* and the bamboo shark *Chiloscyllium punctatum* in their heart and in various organs along the gastrointestinal tract (Figure S8B). Thus, while *Kazald4* is the second-most commonly maintained Kazald gene across jawed vertebrate lineages, a conserved expression profile that could explain this continued preservation remains unknown.

### Kazald1 expression has ancestral ties to the skeleton and teeth

**Figure 4:**
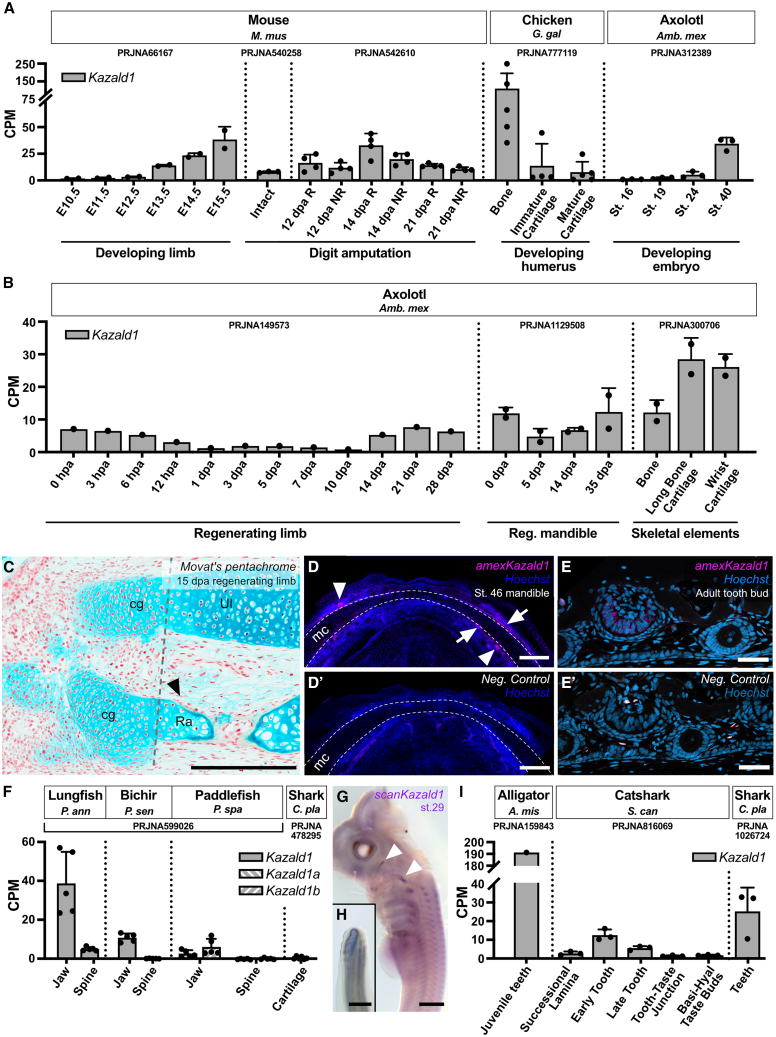
*Kazald1* is expressed during skeletal and tooth development in tetrapods, and potentially in all jawed vertebrates (A) Quantification of *Kazald1* expression in the developing whole limbs of embryonic mice, the intact, regenerating (R), and non-regenerating (NR) digit tips of amputated adult mice, the developing humerus of stage HH36 embryonic chickens, and whole embryos of different developmental stages of the axolotl. (B) Quantification of *Kazald1* expression in adult axolotl during limb and mandible regeneration, and in the intact bone and cartilage of limbs. (C) Movat’s pentachrome staining of 15 dpa juvenile axolotl limb. cg = cartilaginous growth, Ul = ulna, Ra = radius. Scale bar, 500 μm. (D and D′) Maximum Z projection of whole-mount HCR for *Kazald1* and negative control in the lower jaw of stage 46 larval axolotl. Dashed line indicates the location of Meckel’s cartilage (mc). Arrows indicate *Kazald1* expression associated with the ossifying dentary bone. Arrowheads indicate *Kazald1* expression associated with a tooth bud. Scale bars, 250 μm. (E and E′) HCR of *Kazald1* and negative control in a sectioned tooth bud of adult axolotl. Scale bars, 100 μm. (F) Quantification of *Kazald1* expression in the jaw and spine of the lungfish, bichir, and paddlefish, and in the cartilage of the bamboo shark. *Kazald1* was duplicated in the lineage leading to paddlefish with no subsequent loss, creating *Kazald1a* and *Kazald1b*. (G) Whole-mount *in situ* for *Kazald1* in the stage 29 catshark embryo head and upper trunk. Arrowheads indicate expression associated with the pharyngeal arches. Scale bar, 1 mm. (H) Tail tip of embryo in G. Scale bar, 500 μm. (I) Quantification of *Kazald1* expression in the tooth of juvenile alligator, at different stages of tooth development in adult catshark, and teeth of adult bamboo shark. Non-tooth tissue is included as controls for expression in other areas of the jaw of the catshark. PRJ IDs indicate the publicly available RNA-Seq datasets that generated the raw data for the listed tissues. Dots represent biological replicates in examined datasets, and error bars represent standard deviation when calculable. CPM = counts per million, St. = stage, dpa = days post amputation, hpa = hours post amputation. Data are represented as mean ± SD.

Previous investigations have associated *Kazald1* to skeletal development, finding expression in maturing osteoblasts and odontoblasts during bone and teeth mineralization in the mouse.^19^ Thus, we investigated if this expression in skeleton and tooth development may be deeply ancestral, or if it was a later innovation in the lineage leading to mammals, through analyzing published RNA-Seq reads of skeletal and dental tissues from a variety of species of diverse lineages. Starting in the mouse, we examined data from developing forelimbs of embryos and regenerating digits of adults, as both undergo periods of rapid ossification.^51^^,^^52^ The former displayed a clear increase in *Kazald1* expression over limb development (Figure 4A), importantly reflecting RNA *in situ* hybridization experiments from published studies.^19^ Meanwhile, the latter uncovered a peak of *Kazald1* expression in regenerating digit tips (R) at 14 days post amputation (dpa), which was not as striking in non-regenerating digits (NR), and thus likely corresponds to the period of greatest bone reformation during regeneration.^51^ This reinforces the link of *Kazald1* with skeletal ossification, and extends it to adult mouse tissues.

We next examined *Kazald1* expression in RNA-Seq data from other sarcopterygians, and found strong expression of *Kazald1* in the bone of the developing humerus of embryonic chicken (Figure 4A). Furthermore, this gene was expressed specifically in bone and not cartilage, strongly suggesting that, similar to mouse, expression is linked to osteoblasts. Data from axolotl also displayed increased *Kazald1* expression in the final stages of embryonic development, as well as during later stages of limb and jaw regeneration (Figures 4A and 4B), both times in which extensive growth of skeletal elements occurs.^12^^,^^53^

However, in contrast to chicken, axolotl data revealed substantial *Kazald1* expression in both bony and cartilaginous regions of the intact limb. Expression in cartilage could explain why axolotl stage 40 whole embryos already express this gene even though ossification only starts occurring 10–12 days later in the jaw.^54^^,^^55^^,^^56^ Similarly, expression in cartilage could be why *Kazald1* returns to baseline levels in regenerating limbs by 15 dpa, despite regenerated skeletal elements not yet being mineralized (Figure 4C). Finally, axolotl limb chondrocytes were reported to express certain genes considered to be osteoblast-specific in amniotes,^57^ which might include *Kazald1*. Interestingly, though, this may differ between parts of the skeleton, as our *in situ* hybridization chain reaction (HCR) of the embryonic jaw clearly shows expression in the ossifying dentary bone rather than in the chondrocytes of the Meckel’s cartilage (mc) (Figure 4D, white arrows).

Outside tetrapods, examination of RNA-Seq data from lungfish, a non-tetrapod sarcopterygian, and bichir and paddlefish, two non-teleost actinopterygians, revealed a lack of *Kazald1* expression in their spines, but variable amounts in their jaws (Figure 4F). Interestingly, their spines are largely cartilaginous, while their jaws are ossified.^58^^,^^59^^,^^60^^,^^61^^,^^62^^,^^63^^,^^64^ Therefore, the difference in expression between these skeletal elements could suggest that, unlike axolotl but similar to amniotes, *Kazald1* expression in these osteichthyan fish is limited to ossified regions. However, the expression of *Kazald1* across several lungfish tissues (Figure S9A), complicates a conclusive association to skeletogenesis in this species. Despite this, expression within skeletal elements during skeletogenesis, even of those that remain cartilaginous, might still be ancestral to jawed vertebrates, as our whole-mount *in situ* hybridization (WISH) of catshark embryos seemed to associate *Kazald1* with the pharyngeal arches that develop into the jaws (Figure 4G, white arrowheads). However, this expression may instead coincide with certain cranial nerves,^65^ which would match the expression we observed in the adult brain of another catshark species. Furthermore, we did not find it expressed in data generated from lamprey skeletal elements (Figure S7D). Thus, for now, a Kazald gene being expressed within the vertebrate skeleton can only be traced to the ancestor of modern tetrapods.

The variable levels of *Kazald1* in these sarcopterygian and actinopterygian fish jaws could also indicate this gene being expressed in their teeth, the other major mouse tissue that expresses *Kazald1*.^19^ These fish differ greatly in dentition, with lungfish possessing large and continuously growing tooth plates,^66^ bichir exhibiting many conical teeth,^67^ and paddlefish lacking teeth as adults.^68^ Thus, the correlation of *Kazald1* expression with the extent of dentition raises the possibility that the connection to tooth development also exists in these early diverging species. We thus examined published RNA-Seq data of the teeth of several species.

This revealed high expression in alligator teeth, some expression in the early developing tooth of the shark *Scyliorhinus canicula*, and variable expression in the teeth of the shark *Chiloscyllium plagiosum* (Figure 4I). Moreover, our RNA *in situ* hybridization study of *S. canicula* embryos found it expressed in developing caudal dermal denticles (Figure 4H), which express many tooth-associated genes.^69^ Finally, our HCR staining of embryonic and adult axolotl jaws found robust expression within their teeth (Figure 4D, white arrowheads; Figure 4E). Ultimately, these findings suggest an association between *Kazald1* and odontogenesis in tetrapod and chondrichthyan lineages, indicating this connection is likely ancestral to jawed vertebrates.

### Kazald3 is prone to loss, but has potentially replaced Kazald1 in teleost fish

**Figure 5:**
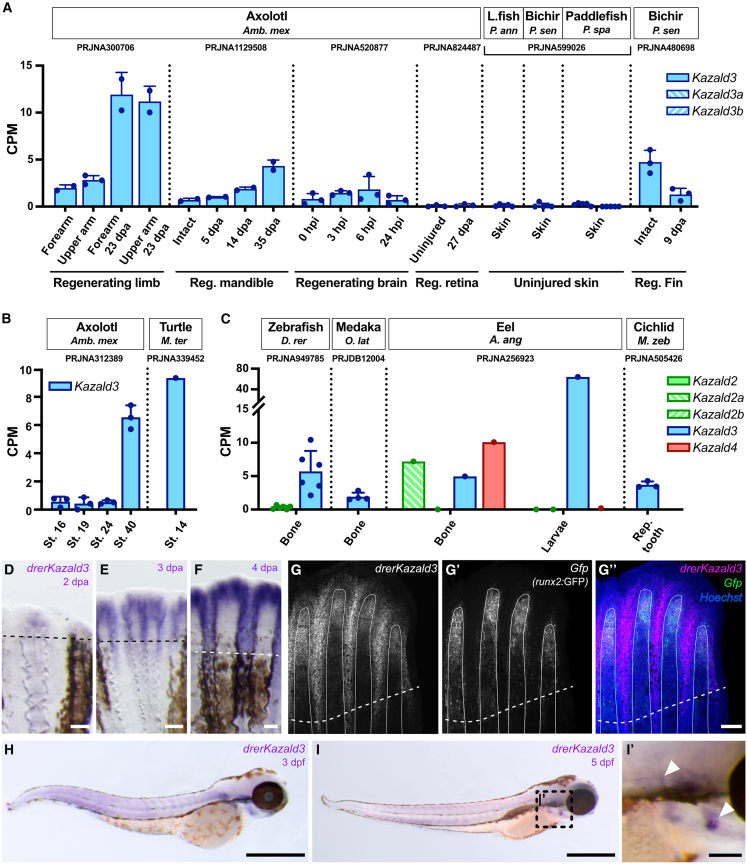
*Kazald3* may have a connection to skeletogenesis and regeneration in teleost fish, but has no clear role in other lineages (A) Quantification of *Kazald3* expression in regenerating limbs, lower jaw, brain, and retina of adult axolotl, the uninjured skin of non-tetrapod/teleost fish, and in regenerating fins of adult bichir. *Kazald3* was duplicated in the lineage leading to paddlefish with no subsequent loss, creating *Kazald3a* and *Kazald3b*. L.fish = Lungfish. (B) Quantification of *Kazald3* expression in whole embryos of developing axolotl and turtle. (C) Quantification of Kazald gene expression in the bone of adult zebrafish, bone of adult medaka, adult bone and the larvae of eel, and the developing tooth of the mbuna cichlid. *Kazald2* was duplicated in the lineage leading to eel with no subsequent loss, creating *Kazald2a* and *Kazald2b*. (D–F) Whole-mount *in situ* hybridization of *kazald3* in regenerating caudal fins of adult zebrafish from 2 to 4 dpa. Dashed line indicates the amputation planes. Scale bars, 100 μm. (G–G″) HCR of *kazald3* (G), *gfp* (G′), and their overlap (G″) in 4 dpa regenerating caudal fin of adult *runx2*:GFP transgenic zebrafish. Dashed line indicates the amputation plane; solid white lines outline individual fin rays. Scale bar, 100 μm. (H and I) Whole-mount *in situ* hybridization of *kazald3* in 3 (H) and 5 (I) dpf zebrafish embryos. Scale bars, 500 μm. (I′) Inset displaying the boxed region of the embryo in I. Arrowheads indicate areas of *kazald3* expression that associate with the locations of ossifying skeletal elements (i.e., the opercle and cleithrum). Scale bar, 100 μm. PRJ IDs indicate the publicly available RNA-Seq datasets that generated the raw data for the listed tissues. Dots represent biological replicates in examined datasets, and error bars represent standard deviation when calculable. CPM = Counts Per Million, St. = stage, dpa = days post amputation, hpi = hours post injury, Reg. = regenerating, Rep. = replaced. Data are represented as Mean ± SD.

In contrast to *Kazald1*, *Kazald3* appeared quite prone to loss, being completely absent in chondrichthyans and commonly lost in sarcopterygians (Figure 2C). This absence in common tetrapod models makes it difficult to identify ancestral expression profiles or determine if it even has one. RNA-Seq data from axolotl barely expressed this gene in any of a variety of tissues, which was also the case in data from the turtle *Malaclemys terrapin* and several non-tetrapod/teleost fish (Figures S9 and S10). Expression in axolotl did slightly increase in the limb and jaw during regeneration, but a similar upregulation was not observed in regenerating brain or retina (Figure 5A). Examination of spatial transcriptomic data of regenerating axolotl limb previously published by our lab^70^ revealed sparse *Kazald3* expression predominantly spread across the limb dermis, which could explain the lack of upregulation in regenerating brain and retina.

Meanwhile, *Kazald3* was only scarcely expressed at most in RNA-Seq data from uninjured skin of other species, which could imply that expression in skin only occurs during regeneration. However, analysis of data from bichir fin found a decrease in *Kazald3* expression during regeneration, and so upregulation during regeneration may instead be specific to axolotls/salamanders. Besides regeneration, *Kazald3* was also lowly expressed in certain embryonic stages of axolotl and turtle (Figure 5B). Thus, a usage for *Kazald3* during development may exist within the few tetrapod species still possessing it, but future study is needed to confirm this. Ultimately, we detected no strong or conserved links between *Kazald3* and any tissue or biological process within the few sarcopterygian species that still maintain it, potentially explaining why this gene is so prone to being lost in this lineage.

In a major departure from these other lineages though, *Kazald3* is the only consistently conserved Kazald gene in teleost fish (Figure 2C). This is also associated with an extremely unusual loss of *Kazald1*, which is otherwise conserved in all other lineages. Due to the ancestral connection of *Kazald1* with skeletogenesis and odontogenesis, we hypothesized that *Kazald3* may have replaced *Kazald1* in these tissues in teleosts. Analysis of published RNA-Seq data from three distantly related teleost fish was inconclusive in linking *Kazald3* with bones (Figure 5C). Supporting our hypothesis, expression levels in intact teleost bones were similar to those of *Kazald1* in adult mouse and axolotl intact bones (Figures 4A and 4B). Furthermore, *Kazald3* was heavily expressed in eel larvae, a stage with bone ossifying in the jaw, operculum, and caudal fin, and preceding extensive bone ossification throughout the body.^71^ However, *Kazald2a* and *Kazald4* being more highly expressed than *Kazald3* in the eel bone could indicate that other and/or multiple Kazald genes were instead associated to the bone in certain teleost fish lineages. This, combined with the overall low expression of *Kazald3* in the adult bones of each fish species, prevented making any conclusions regarding its connection to teleost skeleton development.

We thus examined public single-cell zebrafish gene expression atlases, which revealed clear expression of *kazald3* in osteoblasts, but also in non-osteoblast blastema cells and the basal layer of the wound epidermis in the regenerating fin at 3 dpa.^72^ Our WISH analysis of multiple days of fin regeneration found expression throughout the ray tissue and inter-ray areas of the regenerate, corroborating this single-cell data (Figures 5D–5F). Furthermore, HCR staining of 4 dpa fins found *kazald3* expression in the fin ray overlapped with the area of heavy osteoblast concentration, identified by *green fluorescent protein* (*gfp*) expression driven by a *RUNX family transcription factor 2* (*runx2*) promoter,^73^ supporting a connection with new bone ossification (Figure 5G). To investigate if expression was limited to regenerating tissue, we examined developing zebrafish larvae. Our WISH analysis showed that *kazald3* also overlapped with the region of bone ossification in the developing head at 3- and 5-days post fertilization (dpf) (Figures 5H and 5I), such as in the area of the operculum at 5 dpf (Figures 5I and 5I′).

Thus, our work suggests that *kazald3* has replaced *Kazald1* during bone formation in zebrafish, and potentially across teleosts in general, explaining its uniquely strong conservation within this lineage. We also show that it likely possesses roles outside the skeleton in teleosts, as demonstrated by its expression in non-osteogenic tissue in regenerating fins.

### Kazald2 is expressed during regeneration in most bony vertebrates

Our extensive phylogenetic analysis demonstrated that the regeneration-associated axolotl gene, which prompted this investigation, is *Kazald2*. However, it also uncovered widespread loss of *Kazald2* across lineages (Figure 2C). Such a common loss could indicate *Kazald2* lacked a useful role in these lineages, or was replaced by a different gene(s). Alternatively, its loss could be connected to the loss in regenerative potential of many of these lineages, although it is unclear which loss would have occurred first in this case. To investigate if *Kazald2* has an ancestral connection to regeneration and its correlation to regenerative potential, we analyzed published RNA-Seq data from regenerating tissues of distantly related species that still maintain *Kazald2*.

**Figure 6:**
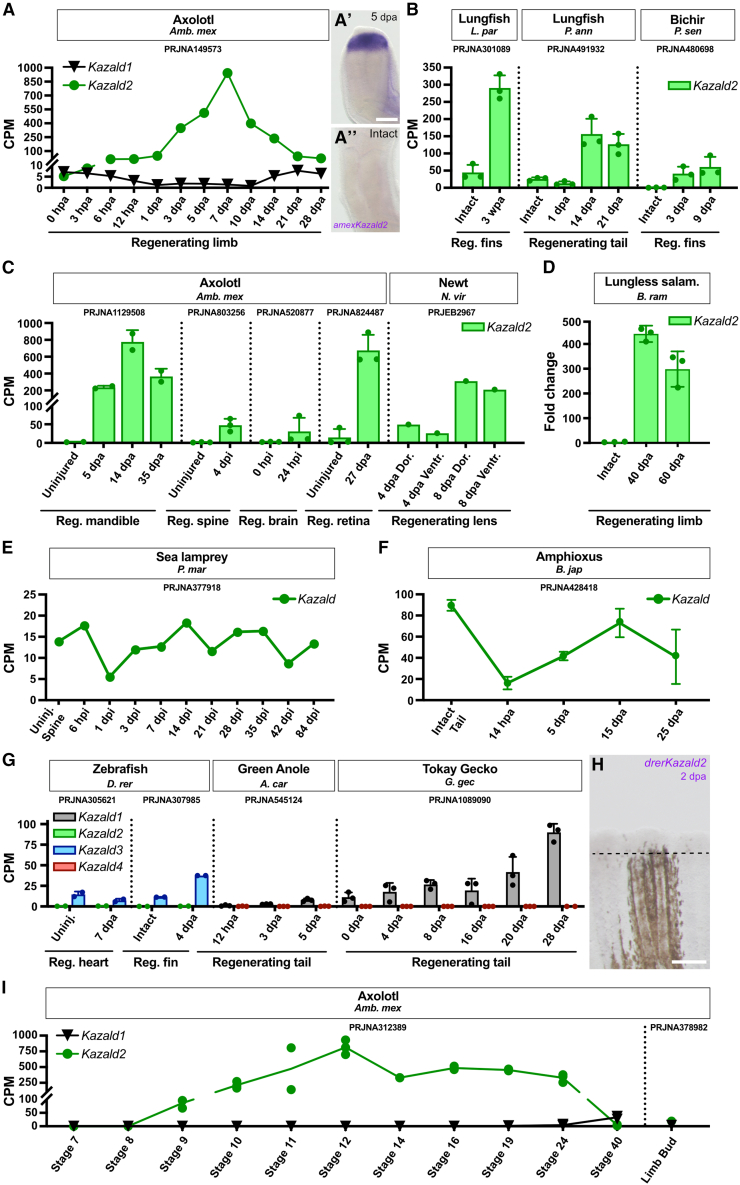
*Kazald2* is expressed during regeneration in species throughout the osteichthyan lineage (A) Quantification of *Kazald1* and *Kazald2* expression over the course of axolotl limb regeneration. (A′ and A″) Whole-mount *in situ* hybridization of *Kazald2* in 5 dpa and intact axolotl limb. Scale bar, 500 μm. (B) Quantification of *Kazald2* expression in the regenerating fins and tail of lungfish and bichir. (C) Quantification of *Kazald2* expression in the regenerating mandible, spine, brain, and retina of axolotl, and the regenerating lens of newt. (D) RT-qPCR examining *Kazald2* expression in the regenerating limbs of the lungless salamander. (E) Quantification of *Kazald* expression over the course of lamprey spine regeneration. (F) Quantification of *Kazald* expression over the course of amphioxus tail regeneration. (G) Quantification of *kazald2* and *kazald3* expression in the regenerating heart and caudal fin of zebrafish, and *Kazald1* and *Kazald4* expression in the regenerating tails of green anole and tokay gecko. (H) Whole-mount *in situ* hybridization of *kazald2* in regenerating caudal fin of adult zebrafish at 2 dpa. Dashed line indicates the amputation planes. Scale bar, 250 μm. (I) Quantification of *Kazald1* and *Kazald2* expression over the course of axolotl embryo development, and in the developing limb bud of axolotl larvae. PRJ IDs indicate the publicly available RNA-Seq datasets that generated the raw data for the listed tissues. Dots represent biological replicates in examined datasets, and error bars represent standard deviation when calculable. CPM = counts per million, hpa = hours post-amputation, dpa = days post-amputation, wpa = weeks post-amputation, hpi = hours post-injury, dpi = days post-injury, Reg. = regenerating, Uninj. = uninjured, Dor. = dorsal, Ventr. = ventral. Data are represented as mean ± SD.

In axolotl, a time course of limb regeneration demonstrated a large increase in expression from negligible levels in the intact limb up to a high peak in the early bud blastema stage at 7 dpa (Figure 6A). This subsequently decreased back to basal levels, reflecting published RNA *in situ* expression patterns.^5^ Examination of data from regenerating fins and tails of non-tetrapod sarcopterygian (two species of lungfish) and non-teleost actinopterygian (one species of bichir) fish revealed these species also greatly upregulate *Kazald2* from intact levels (Figure 6B). This strongly supports a connection of *Kazald2* to regeneration since at least the last common ancestor of all bony vertebrates. Additionally, data from salamanders found that *Kazald2* is highly expressed during regeneration in many bony and soft tissues, including the mandible, spine, and retina (Figure 6C). Expression in the brain was variable, but published HCR staining of *Kazald2*, although referenced as *Kazald1*, has demonstrated weak to strong expression in axolotl regenerating brain from 2 to 7 dpi.^74^

The above-mentioned salamanders, lungfish, and bichir all possess a larval stage, after which most undergo metamorphosis into adulthood.^75^^,^^76^^,^^77^ We wondered if this extended post-embryonic development or maintenance of larval features may enable these species to utilize similar methods of regeneration, such as reactivating developmental pathways that are not restricted to embryonic stages. Thus, we measured *Kazald2* expression via RT-qPCR in regenerating limbs of the direct-developing lungless salamander *Bolitoglossa ramosi*^78^ (Figure 6D). This revealed a similarly large upregulation in the blastema even within a species that is neither paedomorphic nor undergoes metamorphosis.

As a connection of *Kazald2* to regeneration appears independent of the presence of calcified tissue, it could be that a Kazald gene has had this association since before the origin of bony vertebrates. However, since regeneration in cartilaginous fish is extremely limited, we could not determine if this connection existed in the jawed vertebrate ancestor. Thus, we looked further back in evolutionary time by investigating published RNA-Seq data of spine regeneration in the lamprey *P*. *marinus*. Its singular *Kazald* gene did not undergo large or consistent expression changes during regeneration, revealing that this process in lamprey likely occurs without use of a Kazald gene (Figure 6E). Analysis of data from the even more distantly related amphioxus *Branchiostoma japonicum* found that its singular *Kazald* gene was strongly downregulated from high basal levels until near the end of regeneration, demonstrating that this gene is not positively correlated with this process (Figure 6F). Finally, the one species of planaria we found that possesses a *Kazald* gene did not express it at detectable levels in data from either intact or regenerating tissue. Thus, it appears likely that *Kazald2* evolved a connection with regeneration within the lineage leading to bony vertebrates following their split from cyclostomes, but it is unclear if this occurred before or after their split from cartilaginous fish.

However, one lineage of bony vertebrates contains species that still possess *Kazald2* but do not express it during regeneration: teleost fish. Analysis of published RNA-Seq data of intact and regenerating fin and heart of zebrafish revealed almost no expression of *kazald2*, which we confirmed in regenerating fins at 2 dpa via WISH (Figures 6G and 6H). Furthermore, regeneration occurs in teleost species that have lost *Kazald2*, such as medaka.^79^^,^^80^ A possible explanation is that a different Kazald gene is expressed during regeneration instead, which is supported by the large expression of *kazald3* in the blastema and non-bone, inter-ray area of regenerating zebrafish fin (Figure 5D). However, if this is the case, then *Kazald3* in teleost regeneration seems to be more tissue-restricted than *Kazald2* in salamanders, as *kazald3* is not upregulated during zebrafish heart regeneration (Figure 6G). Thus, another possibility is regeneration in teleosts evolved to not need a Kazald gene.

Such a development of Kazald gene-independent regeneration has seemingly occurred in lizards. Analysis of published RNA-Seq data of tail regeneration in two lizard species found that *Kazald4* was mostly non-existent, and that while *Kazald1* continuously increased, its greatest expression in gecko occurred after the period of blastema formation and proliferation (Figure 6G). Thus, its expression pattern better matches the growth of the replacement cartilage rod,^81^ likely reflecting the connection to skeletogenesis we have demonstrated. However, as the tail tissue in that study was always taken from a small region immediately distal to the amputation site,^81^ it could be that *Kazald1* is also expressed by undifferentiated cells in the blastema, but later work is needed to investigate this.

Finally, as a major component of the initial interest in *Kazald2* was its seeming specificity to regeneration, we investigated if it may also be expressed during other biological processes. Analysis of published RNA-Seq data of axolotl embryonic development uncovered that while *Kazald2* is not expressed in the limb bud, matching previous reports,^5^ it is highly expressed from gastrulation (stage 8) to the tailbud stage (stage 24) (Figure 6I). This expression window aligns with that of *Mig30* in *X*. *laevis*,^15^ which we identified to be orthologous to *Kazald2*, potentially suggesting a shared expression pattern. However, this timing also coincides with *X. laevis Kazald1* (then named xIGFBP-rP10) expression during embryogenesis. As axolotl *Kazald1* is not present at this time, the axolotl *Kazald2* expression pattern might match that of *X. laevis Kazald1* in addition to, or instead of, *Mig30*. Thus, future work is still required to determine the exact locations and functions of *Kazald2* expression during axolotl development. Additionally, the naming of *Kazald2* vs. *Mig30* serves as an example of differences in gene identification hindering cross-species research when gene orthology is not easily accessible, as to our knowledge, no comparison of these genes in two of the major amphibian models has been examined.

Outside of jawed vertebrates, we only observed very slight expression of the Kazald genes in hagfish and lampreys during development (Figure S7A). Interestingly, though, the expression window of axolotl *Kazald2* and *X. laevis* Kazald genes might also be shared with amphioxi, where strong upregulation was observed, particularly during neurulation and early larval stages in two species (Figure S7B). However, future work is needed to determine if the expression location and function are similar between these distantly related organisms. Additionally, we saw some expression of *Kazald* in the brittle star *Amphiura filiformis* and sea star *Patiria miniata*, although expression appears to peak during the blastula stage in both species. Meanwhile, tunicates expressed their Kazald gene later on, with expression peaking during rotation (Figure S7C). Finally, Kazald genes have even been found to impact development outside deuterostomes, as published studies of abalones have found their Kazald gene, although referenced as *Igfbp7*, to be involved in metamorphosis.^82^^,^^83^ With such a diversity in developmental expression, the way Kazald genes are used appears to be highly context-dependent, and thus functional studies are necessary to truly understand their roles during embryogenesis in specific species.

## Discussion

Through our work, we uncovered the evolutionary history of a previously undescribed Kazald gene family that appears to have originated in Bilateria. Furthermore, we have shown that this gene family underwent a major expansion in jawed vertebrates, tracing the origin of four paralogous genes to the two-round whole-genome duplication event ancestral to this lineage. Finally, we investigated putative roles for each of these four ohnologs, and determined when their expression profiles most likely arose. Uncharacterized genes and genes with no published descriptions of their roles or functions, such as *Kazald4* and *Kazald3,* respectively, are often overlooked due to the large difficulties incurred by the lack of annotation and prior existing information.^84^ Meanwhile, *Kazald1* and *Kazald2* have had their descriptions limited to just a few species. Thus, our work provides a valuable resource for identifying the processes and/or tissues these genes are connected to in a much wider array of species. Furthermore, it should increase the predictive power concerning the translatability of future findings across species through the determination of how strongly the expression of these genes has been connected to a tissue or biological process over evolutionary time.

### Kazald1 expression during skeletogenesis and odontogenesis is ancestral to jawed vertebrates

The evolution of a mineralized skeleton and other skeletal elements, such as teeth and scales, is one of the most important developments within the jawed vertebrate lineage,^85^^,^^86^ and thus fittingly serves as the basis for the name of the superclass we belong to: Osteichthyes. Exactly how this mineralized skeleton develops has been studied in a wide variety of distantly related species, including the mouse, chicken, salamander, and fish.^57^^,^^87^^,^^88^ Our findings contribute to this cross-species analysis by discovering that *Kazald1* is expressed during both skeletogenesis and odontogenesis across tetrapods. This strongly conserved association of *Kazald1* to this process implies that this gene, and the molecular machinery surrounding it, are likely under heavy selective pressure to remain stable. A probable conclusion is thus that the published findings about the functions of *Kazald1* and the cell types expressing it in mammals^19^^,^^20^ would be applicable across tetrapods as a whole. However, future work to test this hypothesis is required.

The expression of *Kazald1* in odontogenesis is likely even older than the tetrapod lineage, as we identified a conserved expression of it not only in the teeth of the alligator and axolotl, but also in species of sharks. This greatly expands the connection between this gene and teeth from its initial description in mice to now cover all jawed vertebrates. The most parsimonious explanation is that *Kazald1* has had a role in odontogenesis since before the split of the osteichthyan and chondrichthyan lineages, which would make it one of the most ancestral expression domains of any of the four Kazald genes. However, functional examination of how *Kazald1* is being utilized in the teeth of these different species is still required to determine if its exact role and function are equally conserved.

Meanwhile, we show that *Kazald1* expression in the skeletons of non-teleost actinopterygian fish is notably reduced in comparison to tetrapods and other sarcopterygians, and that this gene is completely lost in the teleost fish lineage. The low expression in bichir and paddlefish may be the result of their reduced, and overall cartilaginous, skeletons, which are thought to be derived traits.^59^^,^^62^ Alternatively, the lack of expression, and the eventual loss in teleosts, may be due to the osteichthyan ancestor not actually utilizing *Kazald1* in bone development. Indeed, various key differences in osteoblast gene expression between sarcopterygians and actinopterygians have indicated that certain osteoblast markers likely only developed following the split of these lineages.^89^ A lack of a firmly established usage in ossification would also help explain its varied expression in some species of non-tetrapod/teleost fish, such as *Kazald1b* in paddlefish muscle and *Kazald1* in multiple lungfish tissues. Distinguishing between these possibilities will require work specifically focused on characterizing the expression profiles of osteoblasts in these actinopterygian and sarcopterygian fish to determine when *Kazald1* first started being expressed in the skeleton.

### Kazald2 expression during regeneration is ancestral to bony vertebrates

The other Kazald gene we hypothesize to possess a deeply ancestral role within jawed vertebrates is *Kazald2*, which we showed to be connected to regeneration since at least the last common osteichthyan ancestor. Since appendage regeneration is generally considered to be an ancestral trait that amniotes subsequently mostly lost,^4^ there is hope that findings in other species will one day be translatable to human patients. However, this idea is complicated not just by the vast differences that exist between mammals and the regeneratively capable species currently studied, but also by findings that the mechanisms of regeneration can substantially differ even between more closely related species.

A key example is caudal fin regeneration in zebrafish vs. killifish, in which only a small fraction of differentially expressed genes is shared between the two species.^90^ These species diverged from each other approximately 230 million years ago (mya), much more recently than when humans diverged from zebrafish (∼430 mya), or even from salamanders (∼350 mya).^91^ The larger amount of evolutionary time between humans and these lineages would suggest that even more differences have accrued between them, likely preventing many regeneration-associated genes from functioning the same in the two species. One way to help address this difficulty is by determining just how ancestral and strongly conserved the roles of individual genes are in a given process.

Presumably, if a gene has had a long-standing role across lineages, then the proteins and other molecules that it interacts with to perform that role are also likely to have been similarly conserved.^92^ The ability of a gene to interact with these other molecules may even outlive the gene itself, potentially by it being replaced by a non-orthologous gene or by the other molecules still retaining the interaction site.^93^^,^^94^ We have shown that *Kazald2* upregulation has been most likely linked to regeneration since the bony vertebrate ancestor, and has been conserved in species from both the sarcopterygian and actinopterygian lineages. Thus, this indicates that there is a greater chance that its expression in non-regenerative species could induce some regenerative effects than if this expression during regeneration had been a novel adaptation of the salamander lineage. The loss of *Kazald2* in tetrapods is also correlated with a vast reduction in regenerative potential, although it cannot be determined which of the two might have been the driving force for the other. Finally, the lack of *Kazald2* expression during regeneration in teleost fish such as the zebrafish and medaka, raises the interesting question of whether these species are using entirely different pathways for this process than other bony vertebrates, or if they have replaced *Kazald2* with another gene, such as *Kazald3*.

### Kazald3 has evolved novel expression patterns in teleost fish, but is commonly lost in other lineages

While the previous two Kazald genes both possess clear and deeply ancestral expression profiles within the jawed vertebrate lineage, this does not appear to be the case for *Kazald3*. Most tetrapod lineages have lost this gene, and in the few that still possess it, we were unable to identify a tissue or biological process in which it was expressed above very low levels. Not even the lungfish, which was the sarcopterygian we identified to have the widest diversity of expressed Kazald genes across different tissues, ever expressed *Kazald3* beyond extremely low levels. It is possible that expression profiles for *Kazald3,* which we were unable to uncover, do exist in the sarcopterygians that still retain it, as this gene has been conserved over millions of years. However, the frequent loss of *Kazald3* in many sarcopterygian lineages suggests that the potential roles of this gene within biological tissues or processes may be quite minor, or at least more easily substitutable by other genes.

Although *Kazald3* is commonly lost in sarcopterygians, we discovered that it is actually the most conserved Kazald gene in teleost fish of the actinopterygian lineage. Teleost fish constitute ∼96% of all fish species, and ∼50% of all vertebrates,^95^ and every one of its species that we analyzed possessed *Kazald3*. This remarkably strong maintenance across such a large and diverse clade implies that it has gained an important and overall conserved role within these species, which we have shown could be, at least partially, related to skeletogenesis. Interestingly, this process is what *Kazald1* is connected to in other jawed vertebrates, and thus, the association of *Kazald3* to this process in teleost fish would likely explain the highly unusual loss of *Kazald1* in this lineage. However, further work in other teleost fish species is important to verify the extent of this putative neofunctionalization, as certain species such as the eel express multiple Kazald genes to a similar extent within their bones.

In addition to the overlap that we observed between *Kazald3* and the newly produced bone of regenerating zebrafish fin rays, we also found it to be expressed in a large surrounding region of non-skeletal tissue. This suggests that, in addition to taking on an expression profile similar to *Kazald1* in other lineages, it may potentially have also replaced the use of *Kazald2* within regeneration. This is further supported by our findings that *Kazald2* is not upregulated during zebrafish regeneration, and that this gene has been completely lost in medaka without impairing its ability to regenerate the fin.^79^^,^^80^ However, this requires further testing, as the lack of clear upregulation in the zebrafish regenerating heart indicates that its usage may not be as widespread as that of *Kazald2* in salamanders.

### Kazald4 is highly conserved in gnathostomes without a clear use, but has evolved a novel expression pattern in avian brains

*Kazald4* represents an interesting contrast to *Kazald3*, as it appears to be the second most conserved Kazald gene in jawed vertebrates, when measured by the number of orders still possessing it, but it does not appear to have a correspondingly strongly conserved role. From our examined sarcopterygians, the lungfish did display moderate to strong expression of *Kazald4* in various tissues, such as the lung, jaw, skin, and heart. However, this wide range of expression was not reflected in tetrapods, with most of the species we analyzed not expressing it above low levels in any of the examined tissues or biological processes. This was also the case for actinopterygians, as non-teleost fish, such as the bichir and paddlefish, had very low to nonexistent expression in all of their different tissues. Meanwhile, one teleost fish, the eel, did weakly express *Kazald4* in its bone, but other Kazald genes were also expressed in this tissue at similar levels. Unfortunately, additional analysis in teleost fish was impaired by the most common model species, zebrafish and medaka, not possessing *Kazald4*. Therefore, further examination of other teleost fish that still maintain this gene is needed to discover if a clear usage for *Kazald4* may exist in at least a part of this diverse lineage.

Despite this lack of a clear and conserved expression pattern across the majority of bony vertebrate lineages, we found that birds appear to have developed a strong use for it. Distantly related avian species all expressed high levels of *Kazald4* throughout the brain, which was not observed in any other tetrapod. While our work cannot directly answer what the exact function of this gene in the avian brain is, the high similarity of the four Kazald genes at a genetic and proteomic level makes it plausible that they may perform similar functions at a cellular level. Mainly, *Kazald1* has been functionally characterized and found to promote cell proliferation.^20^ Therefore, it is feasible that *Kazald4* is also involved in cell proliferation in the avian brain. Such an idea is particularly intriguing as, unlike mammals in which adult neurogenesis is limited, birds continue to generate new neurons throughout their lives, with some regions even being described as “hot spots” of neuronal proliferation.^96^^,^^97^ Thus, future studies on whether the centers of *Kazald4* expression correspond to these areas, and especially on what impact its inhibition has on the brain, might provide exciting insights into adult neurogenesis in birds.

### Kazald gene maintenance may be impacted by their 1–2 and α-β relationships

Kazald genes are highly variable in their presence across animal lineages, with clear examples being the differing maintenance of *Kazald3* vs. *Kazald1* in teleosts vs. non-teleosts, the multiple independent losses of *Kazald3* across several orders of turtles, and repeated losses of *Kazald4* throughout the three major lineages of jawed vertebrates: chondrichthyans, actinopterygians, and sarcopterygians. However, some pattern of Kazald gene conservation might still be observable when considering their evolution in terms of the 1–2 and α-β duplications. Namely, we observed that α-β paralogs were always lost before the complete loss of a 1–2 paralog pair, e.g., there were no instances of a lineage maintaining both *Kazald1* (CLGI 1α) and *Kazald3* (CLGI 1β) but losing both *Kazald2* (CLGI 2α) and *Kazald4* (CLGI 2β). Interestingly, we also found that across the three major jawed vertebrate lineages, it was always a Kazald gene from the CLGI 1 segment that was maintained even when all others were lost, regardless of whether it was from the 1α or 1β segment.

As the CLGI 1α/β Kazald genes are seemingly the most strongly conserved ones, it may be that *Kazald1* and *Kazald3* had important but highly similar uses in the α and β ancestors of jawed vertebrates. This could explain their associated expression to the skeleton in tetrapods and teleosts, respectively, and their redundancy would result in the common loss of their α-β pair. Whether *Kazald2* and *Kazald4* represent a similar situation is less clear. We have shown that *Kazald2* has been expressed in regenerating tissues since the bony vertebrate ancestor. However, birds are the only lineage of bony vertebrates we could find that highly expressed *Kazald4*, which was primarily present in the brain. Thus, if *Kazald4* was once expressed during regeneration similar to *Kazald2*, it appears that this expression profile was lost at least in the tetrapod lineage.

Without an apparent function in many of these lineages that would drive purifying selection, the strong conservation of *Kazald4* across jawed vertebrates is perplexing. While a potential explanation is that there actually is a conserved function for *Kazald4* that we were unable to uncover, another fascinating possibility is that the characteristics of the genomic region containing *Kazald4* may be contributing to the preservation of this gene. Several genomic features have been associated with elevated rates of gene loss, including high GC content, high gene density, high densities of repetitive elements, and high nucleotide substitution rates.^98^^,^^99^ Thus, if over the course of jawed vertebrate evolution, the genomic regions containing *Kazald4* have consistently lacked these features, then it may not have been as susceptible to loss. However, addressing what constitutes high gene density or substitution rates in such distantly related species is complicated, since the genomes can differ in size by orders of magnitude and can be changing at very different rates. Future work examining these genomic regions may be able to account for this information to predict if *Kazald4* is likely to possess a functional role we were unable to uncover, or if its persistence in vertebrate genomes could be due to factors beyond the gene itself.

### Possibility for specific Kazald gene replacement with paralogs

Potentially the most interesting question remaining is if specific Kazald genes are able to act in place of their paralogs. Our work has found that all four Kazald genes possess the same protein domains in the same order, and that the three-dimensional structure of the regions containing these domains is all quite similar. Such a high similarity could indicate that the different Kazald paralogs are able to functionally substitute for each other. Indeed, cases of functional shuffling in other gene families have long been known, such as the repeated swaps in expression domains between the Snail family genes *Snail* and *Slug* over the course of vertebrate evolution,^100^ and usages of non-orthologous Hox genes between the mouse and zebrafish for certain developmental patterning.^101^ Further examples of gene substitution continue to be uncovered as searches expand to examine even more species, with notable cases of functional shuffling being found in the Wnt and Fgf families between vertebrates and tunicates.^102^^,^^103^ Our findings that *Kazald3* is expressed in the skeletal tissue and during regeneration in teleost fish, similar to how *Kazald1* and *Kazald2*, respectively, are in tetrapods, support the hypothesis that functional shuffling of Kazald paralogs has likewise taken place within vertebrates in the past. Hopefully, future work will further examine this possibility and investigate if at least certain Kazald genes could act in place of each other.

### Limitations of the study

From our perspective, one limitation of our study is the lack of functional or mechanistic examinations of any of the Kazald genes. While we have found examples of expression within tissues or biological processes for each of the jawed vertebrate Kazald genes, we did not uncover their exact roles. Instead, we relied on the few publications of particular Kazald genes in specific species to predict if those roles extend to other species, as well as to hypothesize potential uses for the other Kazald genes. A second limitation was that single-cell and spatial transcriptomics are only available for a select few of the examined species. These databases could have found more specific expression patterns of the Kazald genes. However, we would not have been able to determine if these expression patterns were also possessed by the majority of the examined species that lack such datasets. Thus, we focused on using bulk RNA-Seq to analyze a wider set of species.

## Resource availability

### Lead contact

Requests for further information, resources and reagents should be directed to the lead contact, Tatiana Sandoval-Guzmán [tatiana.sandoval_guzman@tu-dresden.de].

### Materials availability

Reagents generated in this study can be shared by the lead contact upon request.

### Data and code availability

This study did not generate new RNA-Seq datasets. Generated data (e.g., protein alignment, *in situ* probe sequences, and so forth) and IDs of analyzed existing, publicly available RNA-Seq datasets are provided in the Supplemental Files. This article does not report original code. Any additional information required to reanalyze the data reported in this article is available from the lead contact upon request.

## Acknowledgments

We thank past and current members of the Sandoval-Guzmán lab for their support during the development of this work. We are also grateful to Anja Wagner, Beate Gruhl and Judith Konantz for their dedication to axolotl care. This work was supported by the Light Microscopy Facility and the Histology Facility, both Core Facilities of the CMCB Technology Platform at 10.13039/501100002957Technische Universität Dresden. SDK was supported by the PhD program of the DIGS-ILS and funding from the 10.13039/501100023321TU Dresden Graduate Academy. RA was supported by a 10.13039/501100001659Deutsche Forschungsgemeinschaft Eigene Stelle Grant (AI 214/1-1). We thank Tomoyuki Satonaka at Shima Marineland, Kazuyuki Yamada at Tokai University Marine Science Museum, Daiki Katooka at Enoshima Aquarium, and Hatsune Makino-Itou at the National Institute of Genetics, for catshark embryo sampling. The work at the TU Dresden is co-financed with tax revenues based on the budget agreed by the Saxon Landtag.

## Author contributions

Data curation, formal analysis, methodology, and writing – original draft: S.D.K. Investigation: S.D.K., R.A., B.C.M., C.M.A.G., A.C.L.D., J.P.D., F.K., and S.K. Conceptualization and funding acquisition: S.D.K. and T.S.G. Supervision: S.D.K., S.K., and T.S.G. Visualization: S.D.K., R.A., and T.S.G. Writing – review and editing: all authors. Project administration and resources: T.S.G.

## Declaration of interests

The authors declare no competing interests.

## STAR★Methods

### Key resources table

**Table:** 

REAGENT or RESOURCE	SOURCE	IDENTIFIER
**Antibodies**		

Anti-Digoxigenin-AP, Fab fragments	Roche	Cat#11093274910; RRID: AB_514497

**Biological samples**		

Axolotl limb regenerates	See Experimental models	N/A
Axolotl stage 46 embryos	See Experimental models	N/A
Axolotl mandibles	See Experimental models	N/A
Zebrafish fin regenerates	See Experimental models	N/A
Zebrafish embryos	See Experimental models	N/A
*S. canicula* embryos	See Experimental models	N/A
*B. ramosi* limb regenerates	See Experimental models	N/A

**Chemicals, peptides, and recombinant proteins**		

Benzocaine	Sigma-Aldrich	Cat#E1501-100G
Tricaine	Sigma-Aldrich	Cat#A5040
Tricaine	Sigma-Aldrich	Cat#E10521
Sulfamerazine	Sigma-Aldrich	Cat#S0800
TRIzol	Thermo Fisher Scientific	Cat#15596026
Movat’s Pentachrome	Morphisto	Cat#12057
Hybridization Buffer	Molecular Instruments	Cat#BPH01726
Wash Buffer	Molecular Instruments	Cat#BPW01726
Amplification Buffer	Molecular Instruments	Cat#BAM01826
Hoechst 33258	Abcam	Cat#ab228550
EasyIndex	LifeCanvas Technologies	Cat#EI-500-1.52
ROTI-Histol	Carl Roth	Cat#6640
VECTASHIELD	Vector Laboratories	Cat#H-1000-10
T7 RNA Polymerase	Roche	Cat#RPOLT7-RO
SP6 RNA Polymerase	Roche	Cat#RPOLSP6-RO
Triethanolamine	Sigma-Aldrich	Cat#90278
Acetic Anhydride	Sigma-Aldrich	Cat#320102
Glutaraldehyde Solution	Sigma-Aldrich	Cat#G6257
Blocking Reagent	Roche	Cat#11096176001
BM-Purple	Roche	Cat#11442074001
iQ SYBR Green Supermix	Bio-Rad	Cat#1708880

**Critical commercial assays**		

pGEM-T Easy Vector Systems	Promega	Cat#A1360
Mix2Seq Kit	Eurofins Genomics	Cat#3094-000MSK
RevertAid H Minus First Strand cDNA Synthesis Kit	Thermo Fisher Scientific	Cat#K1632

**Deposited data**		

Reference genomes and transcriptomes	See Data S1	See Data S1
Public RNA-Seq data	See Data S4	See Data S4

**Experimental models: Organisms/strains**		

Axolotl: White (d/d)	N/A	N/A
Zebrafish: WT	N/A	N/A
Zebrafish: runx2:GFP = Tg(Hsa.RUNX2-Mmu.Fos:EGFP)^zf259^	Knopf et al.^73^	https://doi.org/10.1016/j.devcel.2011.04.014
S. canicula: WT	N/A	N/A
B. ramosi: WT	N/A	N/A

**Oligonucleotides**		

oPools Oligo Pools HCR probes	Integrated DNA Technologies	See Data S5
*In situ* hybridization primers	biomers.net	See Data S5
RT-qPCR primers	This paper	See Data S5

**Recombinant DNA**		

pGEMTeasy-scanKazald1	This paper	See Data S5
pGEMTeasy-drerKazald2	This paper	See Data S5
pGEMTeasy-drerKazald3	This paper	See Data S5

**Software and algorithms**		

BLAST+ (version 2.14.1)	Camacho et al.^104^	https://ftp.ncbi.nlm.nih.gov/blast/executables/blast+/
ScanProsite	de Castro et al.^105^	https://prosite.expasy.org/scanprosite/; RRID: SCR_024425
Exonerate (version 2.2.0)	Slater et al.^106^	https://www.ebi.ac.uk/about/vertebrate-genomics/software/exonerate; RRID: SCR_016088
Miniprot (version 0.12-r237)	Li^107^	https://github.com/lh3/miniprot
RAxML-NG (version 1.2.0)	Kozlov et al.^108^	https://github.com/amkozlov/raxml-ng; RRID: SCR_022066
MAFFT (version 7.520)	Katoh and Standley^109^	https://mafft.cbrc.jp/alignment/software/; RRID: SCR_011811
ModelTest-NG (version 0.1.7)	Darriba et al.^110^	https://github.com/ddarriba/modeltest; RRID: SCR_026633
BAli-Phy (version 3.6.0)	Redelings^111^	https://www.bali-phy.org/; RRID: SCR_023976
PhyloBayes-MPI (version 1.9)	Lartillot et al.^112^	https://github.com/bayesiancook/pbmpi
Tracer (version 1.7.2)	Rambaut et al.^113^	https://github.com/beast-dev/tracer
Cutadapt (version 4.9)	Martin^114^	https://cutadapt.readthedocs.io/en/stable/; RRID: SCR_011841
FASTX-Toolkit (version 0.0.14)	N/A	https://github.com/agordon/fastx_toolkit; RRID: SCR_005534
HISAT2 (version 2.2.1)	Kim et al.^115^	https://daehwankimlab.github.io/hisat2/; RRID: SCR_015530
StringTie (version 2.2.1)	Pertea et al.^116^	https://ccb.jhu.edu/software/stringtie/; RRID: SCR_016323
edgeR (version 3.40.2)	Robinson et al.^117^	https://bioconductor.org/packages/release/bioc/html/edgeR.html; RRID: SCR_012802
R (version 4.2.2)	R Core Team^118^	https://www.r-project.org/; RRID: SCR_001905
Salmon (version 1.10.1)	Patro et al.^119^	https://combine-lab.github.io/salmon/; RRID: SCR_017036
ColabFold: AlphaFold2 using MMseqs2 (version 1.5.5)	Mirdita et al.^120^	https://github.com/sokrypton/ColabFold; RRID: SCR_025453
US-align	Zhang et al.^121^	https://aideepmed.com/US-align/
Mol∗ 3D Viewer	Sehnal et al.^122^	https://www.rcsb.org/3d-view
HCR probe generator	Stein et al.^123^	https://hub.docker.com/r/dstein96/probegenerator

### Experimental model and study participant details

#### Animal husbandry

Axolotl husbandry and experimental procedures were performed according to the Animal Ethics Committee of the State of Saxony, Germany. Animals used were selected by their size (snout-to-tail and snout-to-vent lengths). Husbandry was performed in the Center for Regenerative Therapies Dresden axolotl facility adapted from previously published methodology^124^ and according to the European Directive 2010/63/EU, Annex III, Table 9.1. Axolotls were kept in conditions described in ref.^12^

Zebrafish husbandry and experimental procedures were performed according to the animal handling and research regulations of the Landesdirektion Sachsen, Germany (permit numbers: DD24.1-5131/450/4 and 25-5131/564/2 and respective amendments). Husbandry was performed as described in ref.^125^ Adult zebrafish of both sexes were used. The transgenic fish line *runx2*:GFP = Tg(*Hsa.RUNX2-Mmu.Fos*:EGFP)^zf259^ has been described.^73^

Fertilized eggs of the small-spotted catshark (*Scyliorhinus canicula*) were incubated to harvest developing embryos, complying with the guideline defined by the Institutional Animal Care and Use Committee at National Institute of Genetics (Approval ID: R5-14 and R6-13). Embryos were staged according to the developmental stages established in ref.^126^

Wild adult (7–10 cm, snout-to-tail length) *Bolitoglossa ramosi* lungless salamanders were collected from their type locality in the Andes region of Antioquia, Colombia, under the Ministerio del Medio Ambiente contract on access to genetic resources number 118−2015. All experimental procedures were approved by the Institutional Animal Care and Use Committee of the University of Antioquia. These salamanders were collected and kept as described in ref.^127^

#### Axolotl surgery and tissue collection

Axolotls were anesthetized with 0.01% benzocaine solution (Sigma-Aldrich, #E1501-100G) by immersion. Amputations were performed with a scalpel through the forelimb at the mid-radius/ulna level. Following amputation, animals were kept on benzocaine for 15 min and then transferred back to water and allowed to regenerate at 20°C.

Tissue collection was performed by euthanizing animals in a lethal dosage of 0.1% benzocaine solution by immersion for at least 20 min. For HCR, whole stage 46 embryos^54^^,^^56^ were collected and fixed in 4% formaldehyde in 1× phosphate buffered saline (PBS) for 40–60 min and stored in 100% ethanol at −20 °C. For paraffin sectioning and embedding of 15 days post-amputation (dpa) limbs, tissue from three 6 cm (snout-to-tail) animals was fixed in 1× MEMFa (0.1 M MOPS pH 7.4, 2 mM EGTA, 1 mM MgSO4·7H2O, and 3.7% formaldehyde) for a minimum of 3 days, and then decalcified in RNase-free 0.5 M EDTA for 1 week with daily changes of solution. Tissue collection and fixation of adult mandible tissue was performed as in ref.^12^

#### Zebrafish fin clips and tissue fixation

Zebrafish were anesthetized with 0.02% tricaine (Sigma-Aldrich, #A5040) by immersion. Fin clips were performed at 50% of fin length with a scalpel. Animals were transferred to fish water and allowed to regenerate at 28°C.

Fin regenerates and embryos were fixed in 4% paraformaldehyde (PFA) in PBS overnight at 4°C at the indicated times post fin clip and stage, respectively. After fixation, fins and embryos were washed in PBS and dehydrated in methanol for storage at −20°C.

#### *Scyliorhinus canicula* embryo collection

Embryos were extracted from their egg cases by removing the surface layers with a knife and forceps. Embryos were fixed in 4% PFA in PBS overnight at 4°C. After fixation, embryos were dehydrated in PBS/methanol, and stored in 100% methanol at −20°C.

#### *Bolitoglossa ramosi* surgery and tissue collection

Animals were anesthetized with 1% tricaine (Sigma-Aldrich, #E10521) by immersion. Amputations were performed as described in.^127^ Briefly, animals were placed in a Petri dish containing 20 mL tricaine for 4 min. Amputations were performed with microscissors through the forelimb at the mid-humerus level. Protruding bone and muscle were trimmed to obtain a flat wound surface. Following amputation, the wound was rinsed with 1 mL 0.5% sulfamerazine (Sigma-Aldrich, #S0800) to avoid infection. Animals were rinsed with abundant water to remove traces of tricaine, and were transferred to plastic containers and allowed to regenerate at 20°C.

Tissue collection was performed by euthanizing animals in a lethal dosage of 2% tricaine solution by immersion. Tissue was then collected and stored in TRIzol Reagent (Thermo Fisher Scientific, #15596026) until total RNA was extracted following the reagent manufacturer’s protocol.

### Method details

#### Gene nomenclature usage

Published nomenclature guidelines were followed when referencing gene names and symbols within specific species, such as axolotl (e.g., *Kazald1*),^128^ mouse (e.g., *Kazald1*),^129^ zebrafish (e.g., *kazald3*),^130^ and chicken (e.g., *KAZALD1*).^131^ When referencing other species or when discussing genes outside of a species-specific context, the guidelines of the axolotl and mouse were followed.

#### Kazald gene identification in axolotl and other species

The current axolotl (*Ambystoma mexicanum*) genome and transcriptome (RefSeq assembly GCF_040938575.1; WGS project JBEBLI01) were searched through using TBLASTN^104^ (version 2.14.1) run via command line with default parameters using the amino acid sequences of mouse (*Mus musculus*) *Kazald1* and zebrafish (*Danio rerio*) *kazald2* and *kazald3*. Mouse and zebrafish genomes and transcriptomes used for the reciprocal search are listed in Data S1.

The genomes and/or transcriptomes of all other used animal species were downloaded and initially searched through for potential Kazald genes using reciprocal TBLASTN with the amino acid sequences of mouse *Kazald1*, zebrafish *kazald2* and *kazald3*, and the four putative axolotl Kazald genes (Table 1). Animal genomes were chosen to extensively cover the jawed vertebrate lineage, and to provide representatives from major lineages of jawless vertebrates, invertebrate deuterostomes, protostomes, and non-bilaterian animals. Annotated genomes available from NCBI were preferred for selection, but was not a requirement, and are listed in Data S1.

#### Identification of conserved features of putative Kazald genes

The four-exon structure that we considered to be characteristic for vertebrate Kazald genes (Figure S4) and used for subsequent procedures, was identified by checking the reported exon-intron structure of putative Kazald genes in the gff annotation files of examined species. Three protein domains arranged in a specific order were considered to be characteristic of all Kazald genes, which were: (1) Insulin-like growth factor-binding protein domain (Igfbp domain); (2) Kazal-type serine protease inhibitor and follistatin-like domain (Kazal domain); (3) Immunoglobulin-like domain (Ig-like domain). This protein domain order was used for subsequent procedures, and was identified via the Expasy webserver ScanProsite tool^105^ using the putative Kazald gene amino acid sequences listed in available transcriptomes.

#### Identification of unannotated Kazald genes in genomes of species

The genomes of all examined species, when available, were searched through for potential Kazald genes that had not been annotated. This was done in two steps:

1. The genome was searched via TBLASTN using the putative Kazald gene amino acid sequences of the axolotl, gray bichir (*Polypterus senegalus*), West African lungfish (*Protopterus annectens*), and several closely related species to the investigated species, along with the Kazald genes provided in the transcriptome of the investigated species when available. The genome was also searched with the amino acid sequence of the gene *insulin-like growth factor binding protein 7* (*Igfbp7*) of the axolotl and the investigated species, when available, as a control. If there were genomic locations that were more similar to a Kazald gene than they were to *Igfbp7*, and which did not contain any annotated Kazald genes, then these locations were analyzed in Step 2.
2. The identified locations were analyzed via Exonerate^106^ (version 2.2.0) and Miniprot^107^ (version 0.12-r237) using the amino acid sequence of its best match Kazald gene within each of the species used for the TBLASTN search of Step 1. Both programs were run with relaxed parameters, which were modified on a case-by-case basis, to determine if there was a gene sequence in the examined location that could be a Kazald gene. In vertebrates, the sequence was checked for the possession of the characteristic four-exon structure and protein domain order, while in invertebrates only the protein domain order was checked using the Expasy webserver ScanProsite tool. Sequences that fulfilled these criteria were then searched for internal stop codons or frameshift mutations in the Exonerate/Miniprot output files. If none were found, then it was identified as an unannotated Kazald gene that was used in future analysis. Otherwise, the sequence was categorized as a potential pseudogene. Discovered unannotated Kazald genes and Kazald pseudogenes are provided in Data S2 and S3.

#### Editing of annotated Kazald genes

If an annotated Kazald gene within a species was highly dissimilar from the Kazald genes of closely related species, then it was examined to see if the annotation could be incorrect. In vertebrates, this was also done if the annotated gene did not possess the characteristic four-exon structure. Examination was done through two steps:

1. The genomic location containing the dissimilar Kazald gene was analyzed via Exonerate and Miniprot using default parameters with the Kazald genes of closely related species. In vertebrates, the used Kazald genes had to have the characteristic four-exon structure and protein domain order. If a similar sequence to these Kazald genes was found, then the analysis proceeded to Step 2.
2. The genomic locations within the closely related species that contained the Kazald genes used for Step 1 were reciprocally analyzed via Exonerate and Miniprot using default parameters with the dissimilar Kazald gene from Step 1.

If Step 1 uncovered a sequence that was highly similar to the Kazald genes of the closely related species, then that sequence was identified as a putative isoform of the dissimilar Kazald gene, and replaced the dissimilar Kazald gene for use in subsequent analyses. Additionally, if Step 2 failed to discover a sequence in any of the closely related species that was highly similar to the dissimilar Kazald gene, then the dissimilar Kazald gene was determined to likely be an incorrect annotation. Edited Kazald genes are provided in Data S2 and S3.

#### Protein domain prediction

Protein domains were predicted for all genes present in the transcriptomes of several non-bilaterian species using the ps_scan tool available from ScanProsite^105^ run through the command line with default parameters.

#### RNA-Seq read mapping and expression analysis

Downloaded FASTQ files from analyzed BioProjects (listed in Data S4) were trimmed of adapter sequences and low-quality bases using the programs cutadapt^114^ (version 4.9) and fastq_quality_filter from the FASTX-Toolkit (version 0.0.14) (https://github.com/agordon/fastx_toolkit), respectively.

If a genome was available, then the reads were then mapped against it via the program HISAT2^115^ (version 2.2.1). HISAT2 was run through the command line with standard default parameters and a known-splicesite-infile created from the gff annotation file via the hisat2_extract_splice_sites.py script. Transcript quantification was conducted using StringTie^116^^,^^132^ (version 2.2.1) through the command line with standard parameters and the option of assembling novel transcripts. Finally, normalized counts per million (CPM) values for each sample were calculated using the Bioconductor package edgeR^117^ (version 3.40.2), for R^118^ (version 4.2.2).

If a genome was not available, then the reads were mapped against the transcriptome via the program Salmon^119^ (version 1.10.1). Salmon was run through the command line with default parameters. Finally, normalized CPM values for each sample were calculated using the Bioconductor package edgeR for R.

#### Kazald protein 3D structure prediction

Kazald proteins had their 3D structure predicted with AlphaFold2 through the use of the online tool ColabFold: AlphaFold2 using MMseqs2 (version 1.5.5) hosted on a Google Colaboratory Notebook.^120^^,^^133^ Amino acid sequences were used as the query and the tool was run with default parameters.

#### Kazald protein 3D structure alignment and similarity quantification

Kazald proteins were aligned to each other and had their similarity quantified using the Universal Structural alignment (US-align) program through the online webserver hosted by the Zhang lab.^121^ The structural information of proteins, provided within Protein DataBank (.pdb) files, were uploaded and the tool was run with default parameters.

#### Kazald protein 3D structure visualization

The 3D structure of both individual and aligned Kazald proteins were visualized using the Mol∗ 3D Viewer hosted by the RCSB Protein Data Bank.^122^^,^^134^ Visualization was made using the structural information of proteins provided within Protein DataBank (.pdb) files.

#### Paraffin sectioning and Movat’s pentachrome staining

Sample embedding, sectioning and staining of axolotl limbs and adult mandibles was performed by the CMCB Histology Facility, Dresden. Briefly, samples were dehydrated in a series of EtOH in RNase-free water until 100% EtOH, and then embedded in paraffin. Longitudinal sections of 4–5 μm were generated using a microtome. Movat’s Pentachrome (Morphisto, #12057) staining in axolotl limbs was performed according to the manufacturer’s instructions. Imaging was performed using an Olympus OVK automated slide scanner system (UPLFLN 4x/0.13 or UPLSAPO 10x/0.40).

#### Hybridization chain reaction (HCR) staining

Whole mount HCR was performed according to^135^ with some modifications. Briefly, samples were rehydrated through a series of MetOH in RNase-free water and washed three times in PBT (0.1% Tween 20 in PBS). Tissue was then delipidated in Delipidation Solution (200 mM Boric acid, 4% SDS, pH 8.5 in RNAse-free water) for 2 h at 37°C. After three washes in PBT, samples were permeabilized with Permeabilization Solution (0.3M Glycine, 2% Triton X-100, 20% DMSO in PBS) for 1 h at room temperature (RT). Samples were washed again in PBT, incubated in pre-warmed Hybridization Buffer (Molecular Instruments, #BPH01726) for 5 min and then pre-hybridized in new Hybridization Buffer for 30 min at 37°C. After this, tissue was incubated overnight with Hybridization Buffer containing 2 pmol per 500 μL of probe solution. The following day, samples were washed four times with agitation for 15 min with Wash Buffer (Molecular Instruments, #BPW01726) at 37°C and two times for 5 min in 5× SSCT (3M NaCl, 300 mM sodium citrate, 0.1% Tween 20, in water) at room temperature. Pre-amplification was performed for 5 min at RT in Amplification Buffer (Molecular Instruments, #BAM01826), followed by amplification for 16–24 h at RT in Amplification buffer with 30 pmol of each hairpin (Data S5). Finally, tissue was extensively washed in 5× SSCT, incubated overnight with Hoechst 33258 (Abcam, #ab228550) 1:1000 in PBS, and cleared in EasyIndex (LifeCanvas Technologies, #EI-500-1.52) for a minimum of one overnight. Regenerating zebrafish fins and stage 26 axolotl jaws dissected from the embryo were mounted in a glass bottom dish, and then imaged using a Zeiss LSM 980 inverted confocal laser scanning microscope (Plan-apochromat 10x/0.45).

HCR in slides was done according to.^136^ Briefly, slides were dewaxed in ROTI-Histol (Carl Roth, #6640) and rehydrated through a series of EtOH in RNase-free water. After washes in RNAse-free PBS, slides were treated with proteinase K (10 μg/mL in PBS) at 37°C for 10 min. Slides were then washed in RNAse-free water, moved into a humidified chamber containing a solution of 1:1 formamide and 2× SSCT, and pre-hybridized with pre-warmed Hybridization Buffer for 30 min at 37°C. Next, slides were drained from the pre-hybridization solution, covered in Hybridization Buffer containing the probes, protected from drying out with a glass coverslip and incubated overnight at 37°C in the humid chamber. In the following day, the coverslips were removed and the slides were sequentially washed for 15 min at 37°C with 100% HCR Wash Buffer, 75% Wash Buffer/5× SSCT, 50% Wash Buffer/5× SSCT, 25% Wash Buffer/5× SSCT, and 5× SSCT. The slides were then moved to a humidified chamber containing water and pre-amplified with Amplification Buffer for 30 min at RT. Next, slides were covered with a solution of Amplification Buffer containing snap cooled hairpins, covered with parafilm, and incubated for 24 h in a dark humidified chamber at RT. Finally, the slides were extensively washed with 5× SSCT, incubated for 10 min with Hoechst 1:1000 in 5× SSCT, and mounted in VECTASHIELD (Vector Laboratories, #H-1000-10).

Probe sets for axolotl *Kazald1* (*amexKazald1*), zebrafish *kazald3* (*drerKazald3*), and *Gfp* were designed using the HCR probe generator created by the Monaghan Lab^123^ and purchased as oligo pools (oPools Oligo Pools) from Integrated DNA Technologies. Probe sequences can be found in Data S5.

Each HCR was performed in a minimum of 3 different axolotl stage 46 embryos and zebrafish 4 dpa fin regenerates, or 3 different slides corresponding to 3 different animals in axolotl adult mandible tissue.

#### Cloning of RNA probes for *in situ* hybridization

Probes for catshark *Kazald1* (*scanKazald1*), zebrafish *kazald2* and *kazald3* (*drerKazald2* and *drerKazald3*), and axolotl *Kazald2* (*amexKazald2*) were amplified from genomic DNA using primers flanking the first exon of the corresponding gene. Each fragment was then cloned into pGEM-T Easy Vector Systems (Promega, #A1360), according to the manufacturer’s instructions. Constructs were sequenced using the Mix2Seq Kit (Eurofins Genomics, #3094-000MSK) to select for inserts with the correct sequence. Prior to transcription, 10 μg of plasmid were linearized to obtain antisense and sense probes. For synthesizing RNA probes, *in vitro* transcription was carried out using T7 RNA Polymerase (Roche, #RPOLT7-RO) or SP6 RNA Polymerase (Roche, #RPOLSP6-RO), following manufacturer’s instructions. Primer and probe sequences for catshark and zebrafish can be found in Data S5. Primer and probe sequences for axolotl *Kazald2* are from^137^ (listed therein as *Kazald1)*.

#### Whole mount *in situ* hybridization

Whole mount *in situ* hybridization (WISH) was performed using *in vitro* transcribed digoxigenin-labelled antisense RNA probes.

The protocol was adapted from^138^ and performed as in.^137^ Before RNA *in situ* hybridization, samples were dehydrated to 100% MetOH with serial washes of MetOH in RNAse-free water and stored at −20°C. At the start of the protocol, samples were bleached in MetOH +6% H2O2 at RT and fully rehydrated with decreasing concentrations of MetOH in TBST (1× TBS, 0.1% Tween 20) until 100% TBST. Tissues were then washed three times with TBST and treated with 10 μg/mL proteinase K (Pk) in TBST at 37°C. The timing of Pk treatment were as follows: whole mount stage 29 catshark embryos were incubated for 10 min, zebrafish embryos at 3 and 5 dpf were incubated for 15 min, and regenerating zebrafish fins for 20 min. After incubation, samples were washed with TBST and rinsed with 0.1M triethanolamine (Sigma-Aldrich, #90278) in RNAse-free water (TEA) pH 7.5. Tissue was next incubated with freshly prepared 0.1M TEA +1% acetic anhydride (Sigma-Aldrich, #320102) for 10 min and then washed again with TBST. Next, samples were re-fixed with 4% PFA +0.2% glutaraldehyde (Sigma-Aldrich, #G6257) for 20 min and washed with TBST. TBST was removed, and samples were incubated with previously warmed hybridization solution (50% formamide, 5× SSC [3 M NaCl, 300 mM sodium citrate] (pH 5.5), 0.1% Tween 20, 50 μg/mL yeast tRNA, 100 μg/mL heparin, 1× Denhart’s, 0.1% CHAPS, 5 mM EDTA) at 65°C for 4 h. Tissue was incubated with Hybridization Solution containing the RNA probe overnight at 65°C and then washed at 65°C the following day twice with prewarmed 5× SSC (50% formamide, 5× SSC, 0.1% Tween 20), 2× SSC (50% formamide, 2× SSC, 0.1% Tween 20), and 0.2× SSC (0.2× SSC, 0.1% Tween 20) for 30 min each wash. Samples were washed with TNE buffer (10 mM Tris-HCl (pH 7.5), 500 mM NaCl, 1 mM EDTA), treated with RNase (20 μg/mL in TNE buffer) for 15 min, and washed again with TNE buffer. Next, tissue was equilibrated with MABT (100 mM Maleic acid, 150 mM NaCl, 0.1% Tween 20), blocked with MABT/Block (MABT containing 1% Blocking Reagent (Roche, #11096176001)) for 1 h at RT, and incubated with a 1:5000 dilution of alkaline phosphatase-conjugated anti-digoxigenin antibody (Roche, #11093274910) in MABT/Block overnight at 4°C. After extensive washes with MABT at RT, samples were equilibrated in NTMT (100 mM Tris-HCL (pH 9.5), 50 mM MgCl2, 100 mM NaCl, 0.1% Tween 20), and developed at RT in BM-Purple (Roche, #11442074001). Reactions were stopped with PBS and fixed with 4% PFA overnight.

#### RT-qPCR in *Bolitoglossa ramosi*

RNA was extracted from unamputated and regenerating (40 and 60 dpa) forelimb tissue samples from *B. ramosi* lungless salamanders using TRIzol Reagent (Thermo Fisher Scientific, #15596026). RNA was then reverse-transcribed to single-stranded cDNA with reverse transcriptase (Thermo Fisher Scientific, #K1632) in the presence of random hexamer primers, oligoDT primers, and dNTPs for 60 min at 42°C. Expression levels of specific mRNAs were determined by qPCR using gene-specific primer pairs (Data S5), with three technical replicates. Each reaction was performed at a total volume of 10 μL containing 50 ng first-strand cDNA, 5 μL iQ SYBR Green Supermix (Biorad, #1708880), and 0.1 μM of each primer pair, and cycled on a Biorad Real-Time PCR system. Real-time data were analyzed using Biorad software version 2.1. Relative mRNA expression was calculated using the 2 –ΔΔCT method with GAPDH as a cross-sample reference.

### Quantification and statistical analysis

#### Phylogenetic analysis with maximum likelihood

Maximum likelihood phylogenetic trees were created from amino acid sequences of the whole and protein-domain containing regions of putative Kazald genes using RAxML-NG^108^ (version 1.2.0). The peptide sequences were first aligned using MAFFT^109^ (version 7.520), with the L-INS-i alignment setting and default parameters. Alignments are provided in Data S6 and S7. The JTT+I+G4 or JTT+R4 substitution models were used depending on the gene, which were chosen via ModelTest-NG^110^^,^^139^ (version 0.1.7). RAxML-NG was run based on commands described in the tutorial page of the program wiki (github.com/amkozlov/raxml-ng/wiki/Tutorial). A convergence cutoff of 1% was set to determine the number of bootstraps to run.

#### Phylogenetic analysis with Bayesian inference

Bayesian inference phylogenetic trees were created from the whole amino acid sequences of putative Kazald genes using BAli-Phy^111^ (version 3.6.0) and PhyloBayes-MPI^112^ (version 1.9). BAli-Phy was run using the following commands based on the type of analysis.

Analysis of Kazald gene family in all species (6 chains were run): bali-phy Kazald_Peptides.fa -S jtt+Rates.free[n=4] --set infer-ambiguous-observed=true -n Kazald.

Analysis of one specific gene family in limited species (4 chains were run): bali-phy Gene_Peptides.fa -S jtt+Rates.free[n=4] --set infer-ambiguous-observed=true -n GeneName.

Analysis of the syntenic blocks of limited species through partitioned analysis (4 chains were run): bali-phy Adra1_Peptides.fa FgfD_Peptides.fa Gfra_Peptides.fa Kazald_Peptides.fa Lrrtm_Peptides.fa -S jtt+Rates.free[n=4] -n SyntenicBlocks.

The JTT+R4 substitution model was used for all runs, as it was chosen as either the best or second-best model for all genes via ModelTest-NG. BAli-Phy was run until the chains converged, which was determined using the included bp-analyze script, and the program Tracer^113^ (version 1.7.2).

PhyloBayes using the CAT-GTR model was run with the same protein-domain containing region protein alignment file (Data S6) as RAxML. 3 chains were run, each with 8 parallel processes, for 44,000 cycles, saving every 10 cycles. Convergence was confirmed and the consensus tree was generated using the included bpcomp tool with a burn-in of 400.

## Supplemental information

Document S1. Figures S1–S10, Tables S1 and S2, and Supplemental References

Data S1. List of used genomes and transcriptomes used for analyses, related to Figures 1–6

Data S2. Nucleotide sequences of all Kazald genes used for analyses, related to Figures 1 and 2

Data S3. Amino acid sequences of all Kazald genes used for analyses, related to Figures 1 and 2

Data S4. List of publicly available RNA-Seq datasets that were downloaded for use in analyses; includes BioProject IDs and associated publications (Supporting References), related to Figures 3–6

Data S5. List of primers and probes sequences used in experiments, related to Figures 4–6 (A) Primer and probe sequences used for WISH. (B) Probe sequences for axolotl HCR. (C) Probe sequences for zebrafish HCR. (D) Probe sequences for HCR against *Gfp*. (E) Primer sequences for *Bolitoglossa ramosi* RT-qPCR.

Data S6. Protein alignment file used for RAxML tree generation (Figure 1) and PhyloBayes tree generation (Figure S2), related to Figure 1

Data S7. Protein alignments used for RAxML tree generation (Figures S1 and S3), related to Figure 1

Data S8. FPKM values corresponding to all figures, related to Figures 3–6 (A) FPKM values for Figure 3. (B) FPKM values for Figure 4. (C) FPKM values for Figure 5. (D) FPKM values for Figure 6. (E) FPKM values for Figure S6. (F) FPKM values for Figure S7. (G) FPKM values for Figure S8. (H) FPKM values for Figure S9. (I) FPKM values for Figure S10.
