# Supplementary material for: Differential fates of Kazald gene quartet: Ancestral roles in skeletogenesis and regeneration to putative innovations in fish and birds
Source: iScience. 2026 Feb 10;29(3):114934. doi: 10.1016/j.isci.2026.114934 (PMC12955574; doi:10.1016/j.isci.2026.114934)
Supplement: Document S1. Figures S1–S10, Tables S1 and S2, and Supplemental References [file mmc1.pdf]

iScience, Volume *29*

Supplemental information

Differential fates of Kazald gene quartet:

Ancestral roles in skeletogenesis and regeneration

to putative innovations in fish and birds

Sean D. Keeley, Rita Aires, Belfran Carbonell Medina, Claudia Marcela Arenas-Gómez, Alejandra Cristina López-Delgado, Jean Paul Delgado, Franziska Knopf, Shigehiro Kuraku, and Tatiana Sandoval-Guzmán

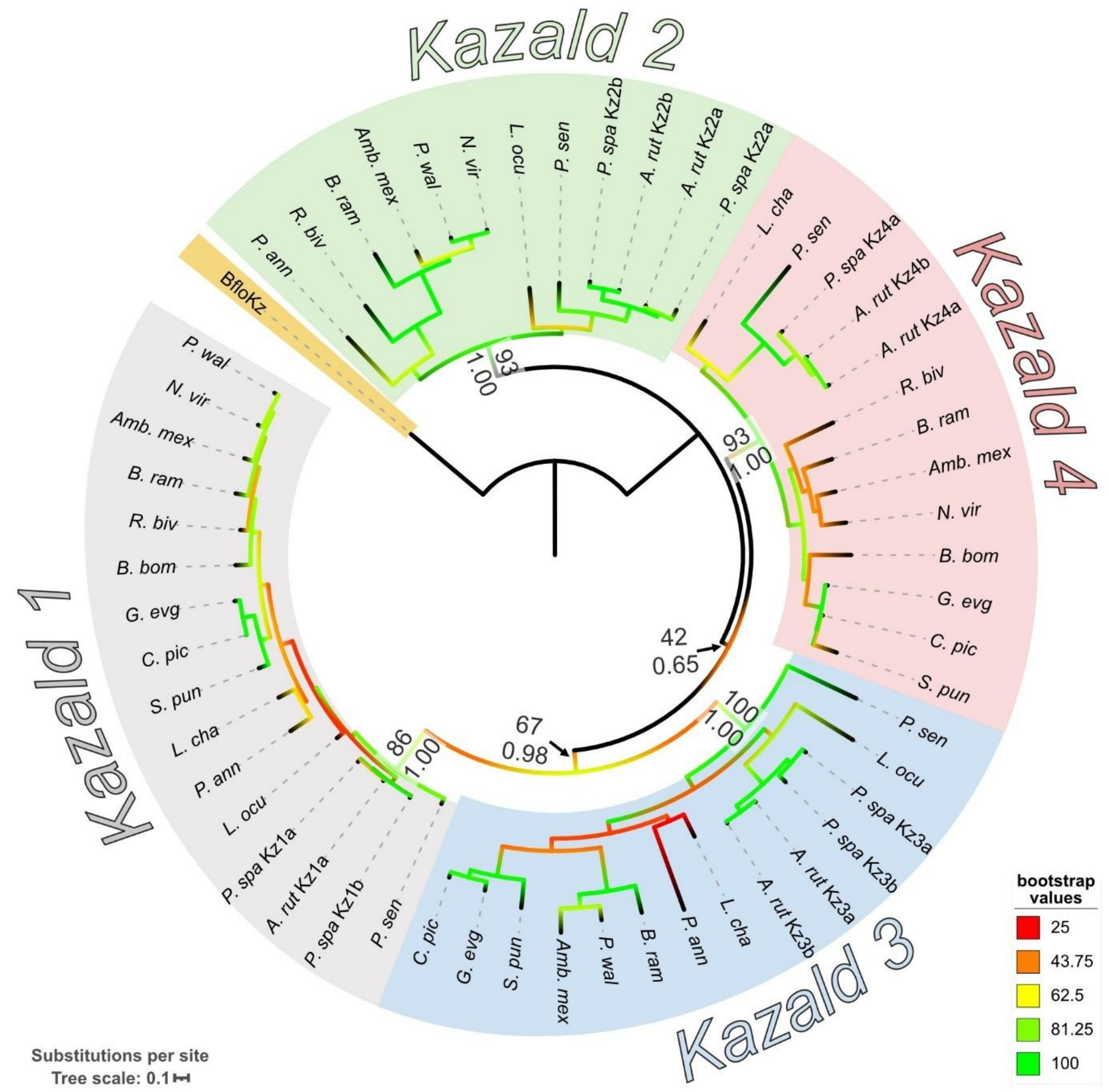

Figure S1. Phylogenetic tree of Kazald genes from a subset of species strengthens the support of the four distinct clades in vertebrates, related to Figure 1.
Displayed consensus tree was generated via RAxML using amino acid sequences. Branch colors reflect the bootstrap values of each node, and do not correspond to branch support. Support values are shown for the nodes basal to the four Kazald clades. Layout of the support values are: ML bootstrap support via RAxML (top), BI posterior probabilities via BAli-Phy (bottom). Abbreviated species names are listed in File S1. Kazald a and b versions created by whole genome duplications that occurred after the 2R-WGD event in the Acipenseriformes order of fish are marked next to the abbreviated species name. Scale bar corresponds to mean number of amino acid substitutions per site.

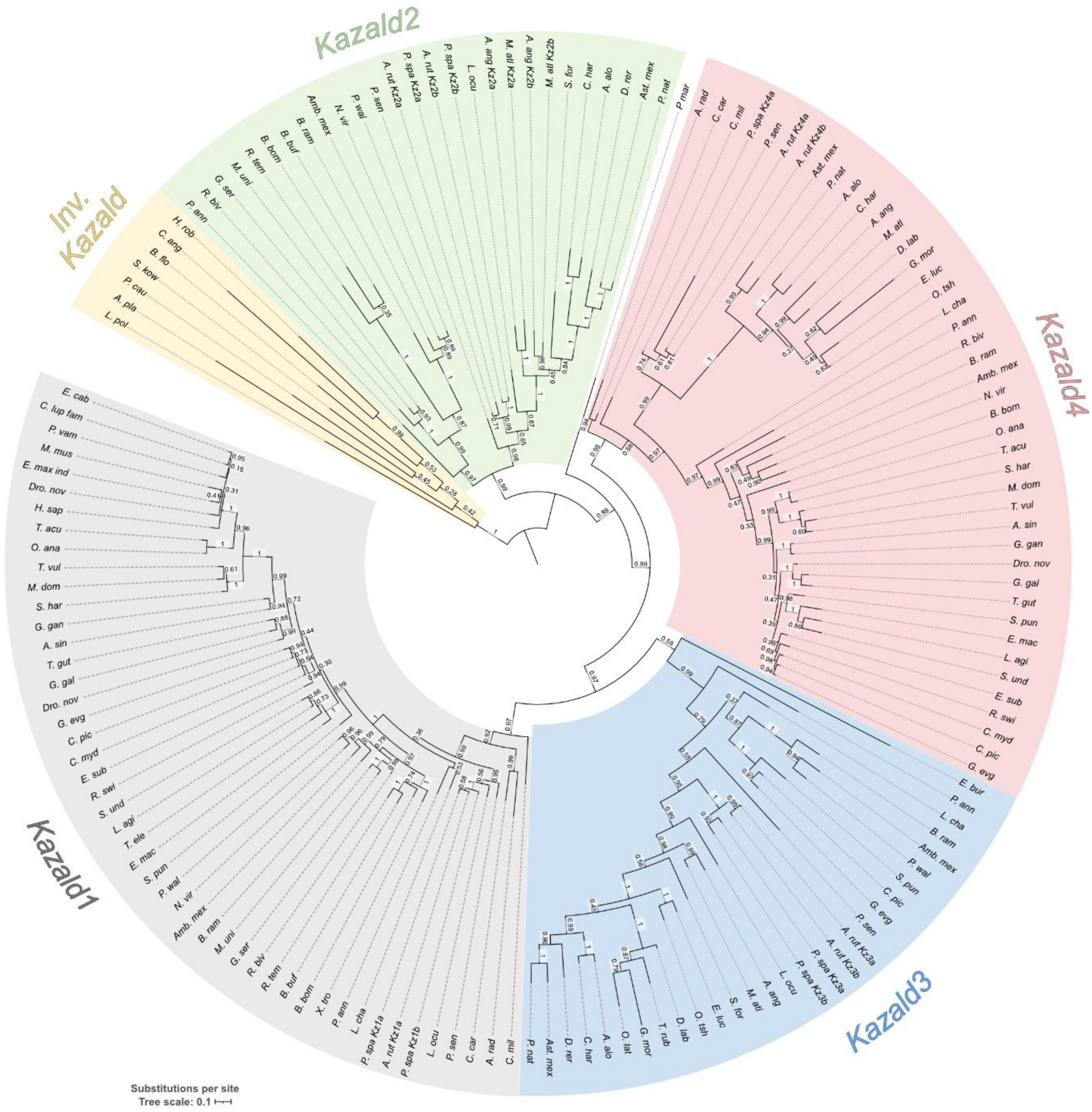

Figure S2. Phylogenetic tree of Kazald genes made with PhyloBayes supports separation of cyclostome Kazald genes, related to Figure 1.
Displayed consensus tree was generated via PhyloBayes using amino acid sequences. Support values are BI posterior probabilities. Abbreviated species names are listed in File S1. Kazald a and b versions created by whole genome duplications that occurred after the 2R-WGD event in the Acipenseriformes order of fish are marked next to the abbreviated species name. Scale bar corresponds to mean number of amino acid substitutions per site.

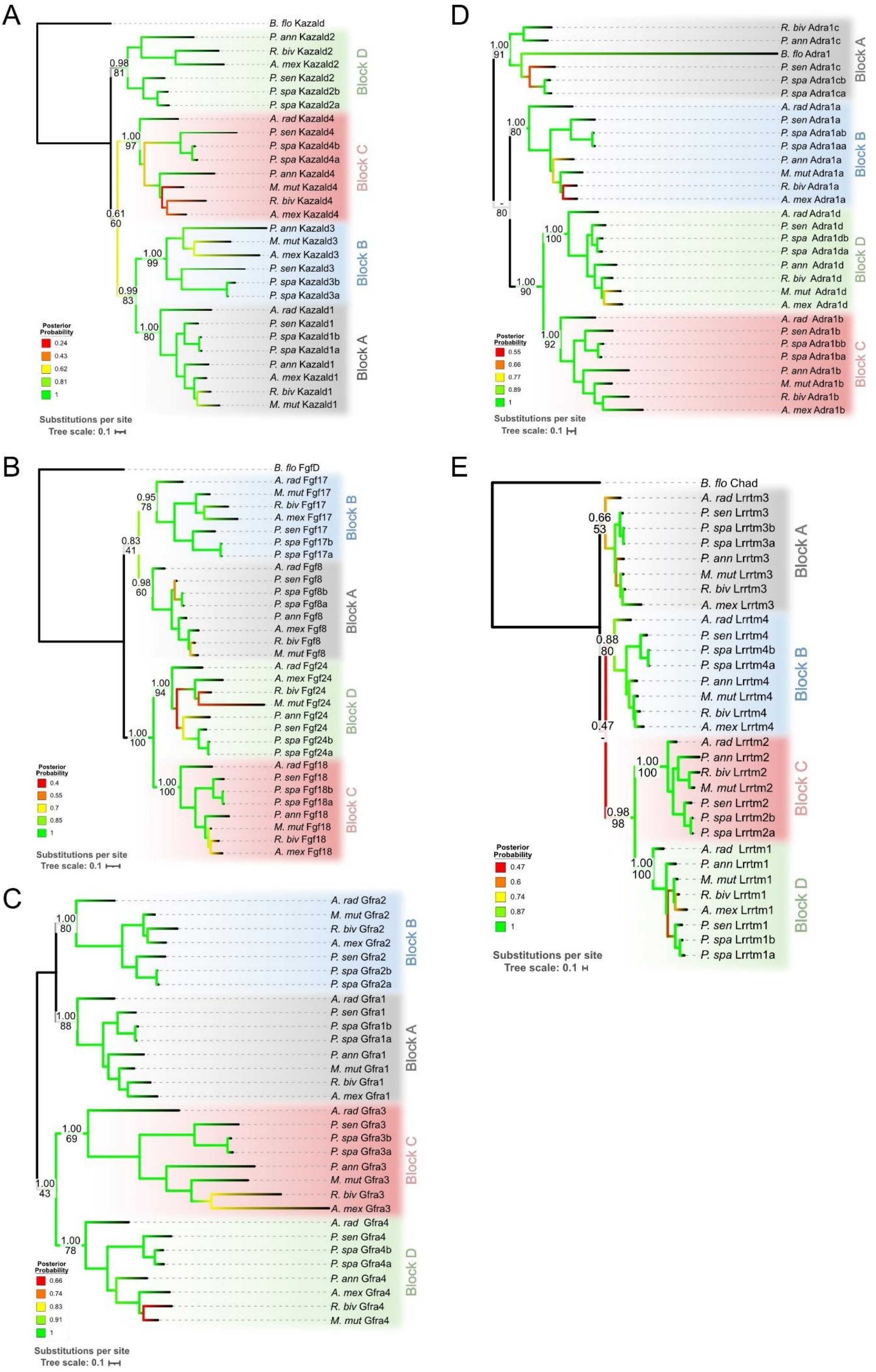
A
B. flo Kazald
P. ann Kazald2
R. biv Kazald2
A. mex Kazald2
P. sen Kazald2
P. spa Kazald2b
P. spa Kazald2a
A. rad Kazald4
P. sen Kazald4
P. spa Kazald4b
P. spa Kazald4a
P. ann Kazald4
M. mut Kazald4
R. biv Kazald4
A. mex Kazald4
P. ann Kazald3
M. mut Kazald3
A. mex Kazald3
P. sen Kazald3
P. spa Kazald3b
P. spa Kazald3a
A. rad Kazald1
P. sen Kazald1
P. spa Kazald1b
P. spa Kazald1a
P. ann Kazald1
A. mex Kazald1
R. biv Kazald1
M. mut Kazald1
Block D
Block C
Block B
Block A
0.98
81
1.00
97
0.61
60
1.00
99
0.99
83
1.00
80
Posterior Probability
0.24
0.43
0.62
0.81
1
Substitutions per site
Tree scale: 0.1
B
B. flo FgfD
A. rad Fgf17
M. mut Fgf17
R. biv Fgf17
A. mex Fgf17
P. sen Fgf17
P. spa Fgf17b
P. spa Fgf17a
A. rad Fgf8
P. sen Fgf8
P. spa Fgf8b
P. spa Fgf8a
P. ann Fgf8
A. mex Fgf8
R. biv Fgf8
M. mut Fgf8
A. rad Fgf24
A. mex Fgf24
R. biv Fgf24
M. mut Fgf24
P. ann Fgf24
P. sen Fgf24
P. spa Fgf24b
P. spa Fgf24a
A. rad Fgf18
P. sen Fgf18
P. spa Fgf18b
P. spa Fgf18a
P. ann Fgf18
M. mut Fgf18
R. biv Fgf18
A. mex Fgf18
Block B
Block A
Block D
Block C
0.95
78
0.83
41
0.98
60
1.00
94
1.00
100
1.00
100
Posterior Probability
0.4
0.55
0.7
0.85
1
Substitutions per site
Tree scale: 0.1
C
A. rad Gfra2
M. mut Gfra2
R. biv Gfra2
A. mex Gfra2
P. sen Gfra2
P. spa Gfra2b
P. spa Gfra2a
A. rad Gfra1
P. sen Gfra1
P. spa Gfra1b
P. spa Gfra1a
P. ann Gfra1
M. mut Gfra1
R. biv Gfra1
A. mex Gfra1
A. rad Gfra3
P. sen Gfra3
P. spa Gfra3b
P. spa Gfra3a
P. ann Gfra3
M. mut Gfra3
R. biv Gfra3
A. mex Gfra3
A. rad Gfra4
P. sen Gfra4
P. spa Gfra4b
P. spa Gfra4a
P. ann Gfra4
A. mex Gfra4
R. biv Gfra4
M. mut Gfra4
Block B
Block A
Block C
Block D
1.00
80
1.00
88
1.00
69
1.00
43
1.00
78
Posterior Probability
0.66
0.74
0.83
0.91
1
Substitutions per site
Tree scale: 0.1
D
R. biv Adra1c
P. ann Adra1c
B. flo Adra1
P. sen Adra1c
P. spa Adra1cb
P. spa Adra1ca
A. rad Adra1a
P. sen Adra1a
P. spa Adra1ab
P. spa Adra1aa
P. ann Adra1a
M. mut Adra1a
R. biv Adra1a
A. mex Adra1a
A. rad Adra1d
P. sen Adra1d
P. spa Adra1db
P. spa Adra1da
P. ann Adra1d
R. biv Adra1d
M. mut Adra1d
A. mex Adra1d
A. rad Adra1b
P. sen Adra1b
P. spa Adra1bb
P. spa Adra1ba
P. ann Adra1b
M. mut Adra1b
R. biv Adra1b
A. mex Adra1b
Block A
Block B
Block D
Block C
1.00
91
1.00
80
-
80
1.00
100
1.00
90
1.00
92
Posterior Probability
0.55
0.66
0.77
0.89
1
Substitutions per site
Tree scale: 0.1
E
B. flo Chad
A. rad Lrrtm3
P. sen Lrrtm3
P. spa Lrrtm3b
P. spa Lrrtm3a
P. ann Lrrtm3
M. mut Lrrtm3
R. biv Lrrtm3
A. mex Lrrtm3
A. rad Lrrtm4
P. sen Lrrtm4
P. spa Lrrtm4b
P. spa Lrrtm4a
P. ann Lrrtm4
M. mut Lrrtm4
R. biv Lrrtm4
A. mex Lrrtm4
A. rad Lrrtm2
P. ann Lrrtm2
R. biv Lrrtm2
M. mut Lrrtm2
P. sen Lrrtm2
P. spa Lrrtm2b
P. spa Lrrtm2a
A. rad Lrrtm1
P. ann Lrrtm1
M. mut Lrrtm1
R. biv Lrrtm1
A. mex Lrrtm1
P. sen Lrrtm1
P. spa Lrrtm1b
P. spa Lrrtm1a
Block A
Block B
Block C
Block D
0.66
53
0.88
80
0.47
-
1.00
100
0.98
98
1.00
100
Posterior Probability
0.47
0.6
0.74
0.87
1
Substitutions per site
Tree scale: 0.1

Figure S3. Bayesian inference trees of each of the multigene families chosen for the partitioned analysis of the Syntenic Blocks demonstrate no discordant topologies, related to Figure 2. Displayed trees were generated via BAli-Phy using amino acid sequences. Branch colors indicate the BI posterior probability at each node. Support values are shown for the nodes basal to the four Syntenic Blocks. Layout of the support values are: BI posterior probabilities via BAli-Phy (top), ML bootstrap support via RAxML (bottom). Scale bars correspond to mean number of amino acid substitutions per site.

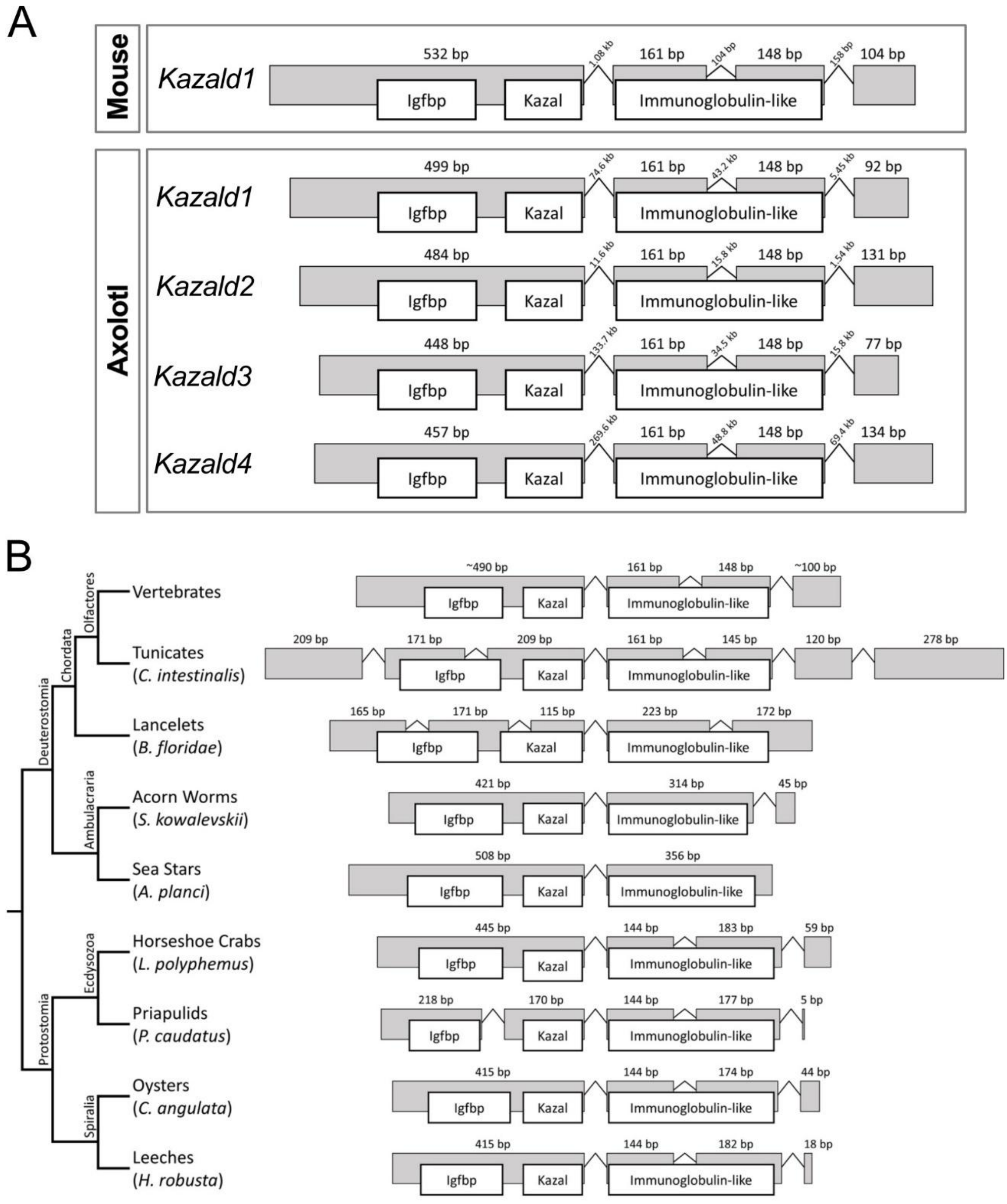

Figure S4. The four jawed vertebrate Kazald genes have a consistent genomic structure, which is also present in protostomes but not invertebrate deuterostomes, related to Figure 2.
Exons are colored in gray and have their relative sizes scaled, introns are represented by bent lines and do not have their sizes scaled. Identity and location of the protein domains are represented by the overlapping white boxes, and are scaled to the size of the exons. (A) Exon-intron structure and protein domain location comparison between mouse *Kazald1* and the four axolotl Kazald genes. (B) Overview of the Kazald gene exon-intron structures and protein domain locations across bilaterians. Abbreviations: bp = base pairs, kb = kilobase.

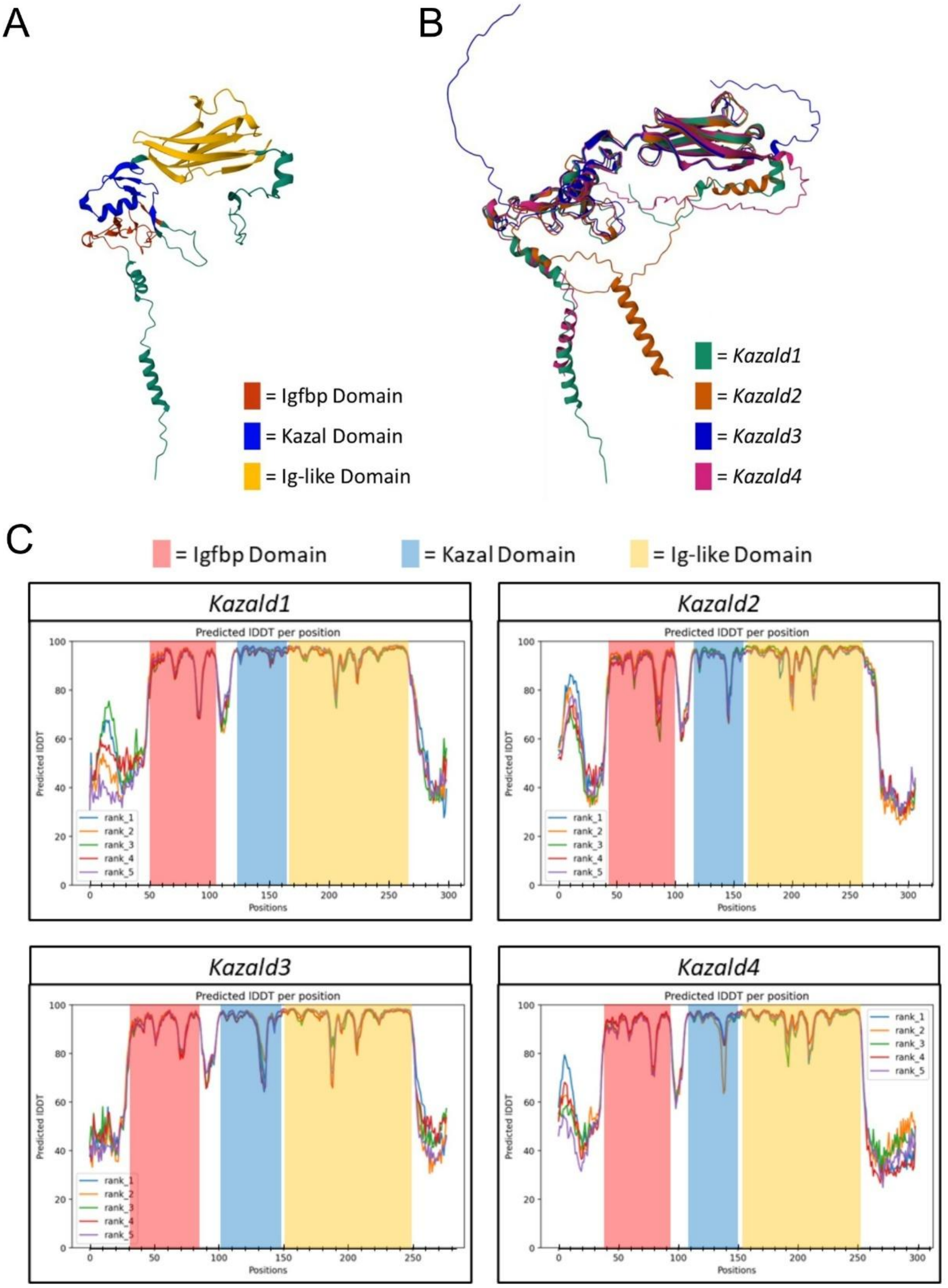

D

TM-score	*Kazald1*	*Kazald2*	*Kazald3*	*Kazald4*
Kazald1		0.94	0.91	0.96
Kazald2	0.94		0.92	0.91
Kazald3	0.91	0.92		0.91
Kazald4	0.96	0.93	0.93	

E

Sequence Identity; NW Score (upper) / Sequence Similarity; Percentage of Gaps (lower)	*Kazald1*	*Kazald2*	*Kazald3*	*Kazald4*
Kazald1		44.7%; 561.0	52.3%; 651.0	58.1%; 707.0
Kazald2	63.5%; 0.9%		43.2%; 501.0	48.9%; 596.0
Kazald3	67.7%; 1.8%	59.0%; 2.7%		51.8%; 629.5
Kazald4	71.0%; 0.9%	67.6%; 1.8%	67.3%; 2.7%	

Figure S5. The regions of each Kazald gene that contain the three protein domains have similar 3D structures, related to Figure 2.

(A) Schematic of the protein structure of axolotl *Kazald1* with the three protein domains highlighted. (B) Schematic of how the four axolotl Kazald proteins best overlap with each other. (C) Graphs depicting the level of confidence that AlphaFold2 has in the predicted protein structure, expressed as the predicted local distance difference test (Predicted lDDT) per position. Five structures for each Kazald gene were predicted and then ranked through AlphaFold2, with the "rank_1" prediction of each one used for all subsequent analysis. (D) Template modeling score (TM-score) of the superimposition of the Protein Domain Containing Regions (PDCRs) of the Kazald gene of the row against the Kazald gene of the column via US-align. As the TM-score is normalized by sequence length, the mirrored TM-scores can be different from each other. A TM-score of 1 equates to the two structures being 100% identical, a TM-score above 0.9 means the structures are nearly identical to one another [S1], (E) Amino acid sequence identity, sequence similarity, percentage of gaps, and NW Score of the PDCRs of two Kazald genes compared against each other via the Needleman–Wunsch algorithm.

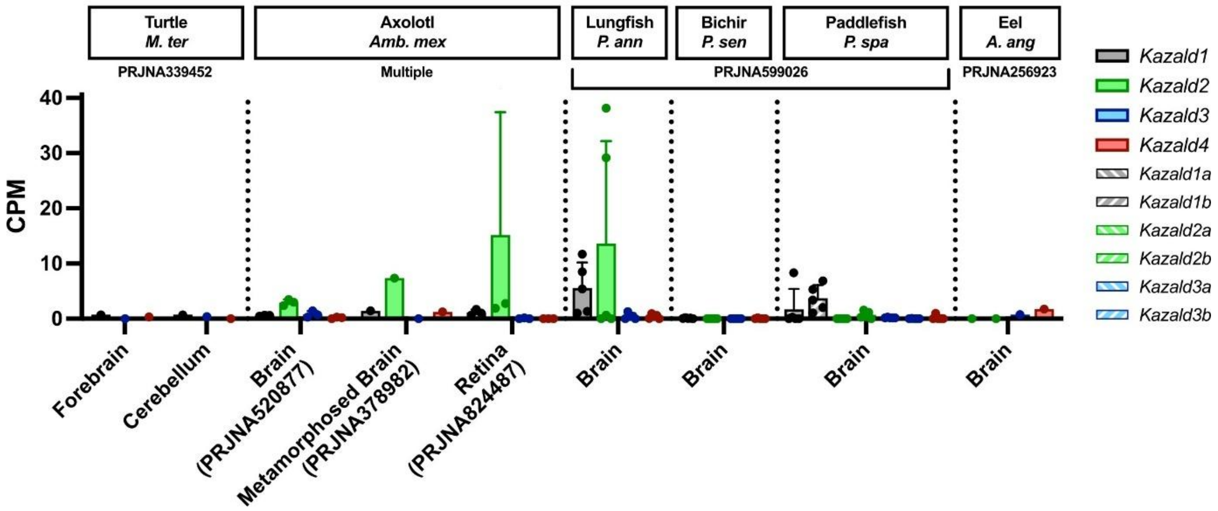

Figure S6. Non-avian species lack a strong and consistent expression of any Kazald gene in their brains, related to Figure 3.

Quantification of the expression of all maintained Kazald genes of a species in different parts of their brains, and eye tissue when available. *Kazald1*, *Kazald2*, and *Kazald3* were duplicated in the lineage leading to paddlefish with no subsequent losses, creating a and b versions of these genes. *Kazald2* was duplicated in the lineage leading to eel with no subsequent loss, creating a *Kazald2a* and *Kazald2b*. PRJ IDs indicate the publicly available RNA-Seq datasets which generated the raw data for the listed tissues. Dots represent biological replicates in examined datasets, error bars represent standard deviation when calculable. CPM = Counts Per Million. Data are represented as Mean ± SD.

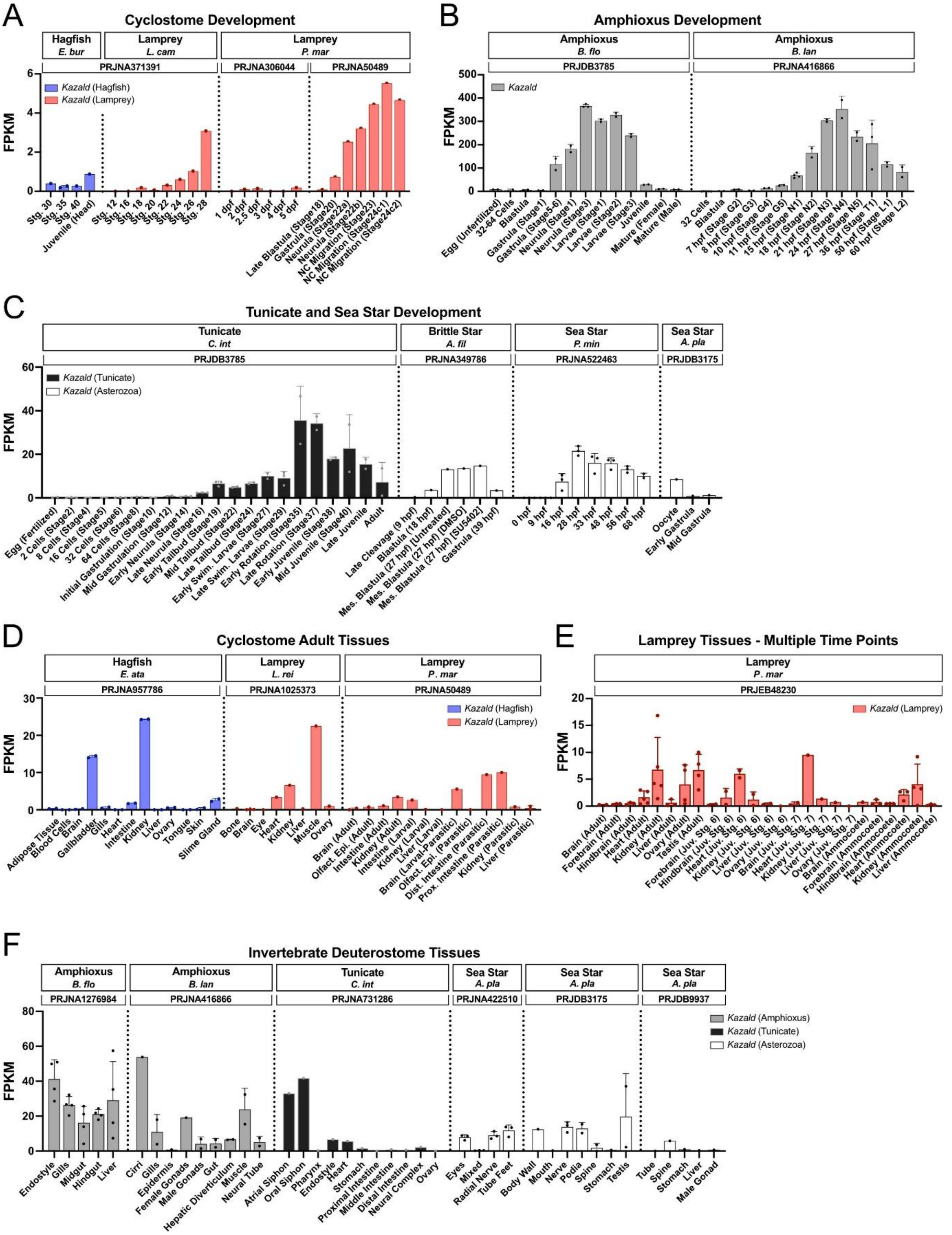

Figure S7. Kazald gene expression during development and in various tissues of non-gnathostome deuterostomes, related to Figures 3, 4, and 6.

(A) Quantification of the expression of Kazald genes during development in hagfish and two species of lamprey. (B) Quantification of *Kazald* expression during development in two species of amphioxi. (C) Quantification of *Kazald* expression during development in tunicate, brittle star, and two species of sea star. (D) Quantification of the expression of Kazald genes in various tissues of hagfish and two species of lamprey. (E) Quantification of *Kazald* expression in various tissues of lamprey at different life stages. (F) Quantification of *Kazald* expression in various tissues of tunicate, sea star, and two species of

amphioxi. PRJ IDs indicate the publicly available RNA-Seq datasets which generated the raw data for the listed tissues. Dots represent biological replicates in examined datasets, error bars represent standard deviation when calculable. FPKM = Fragments Per Kilobase per Million. Data are represented as Mean ± SD.

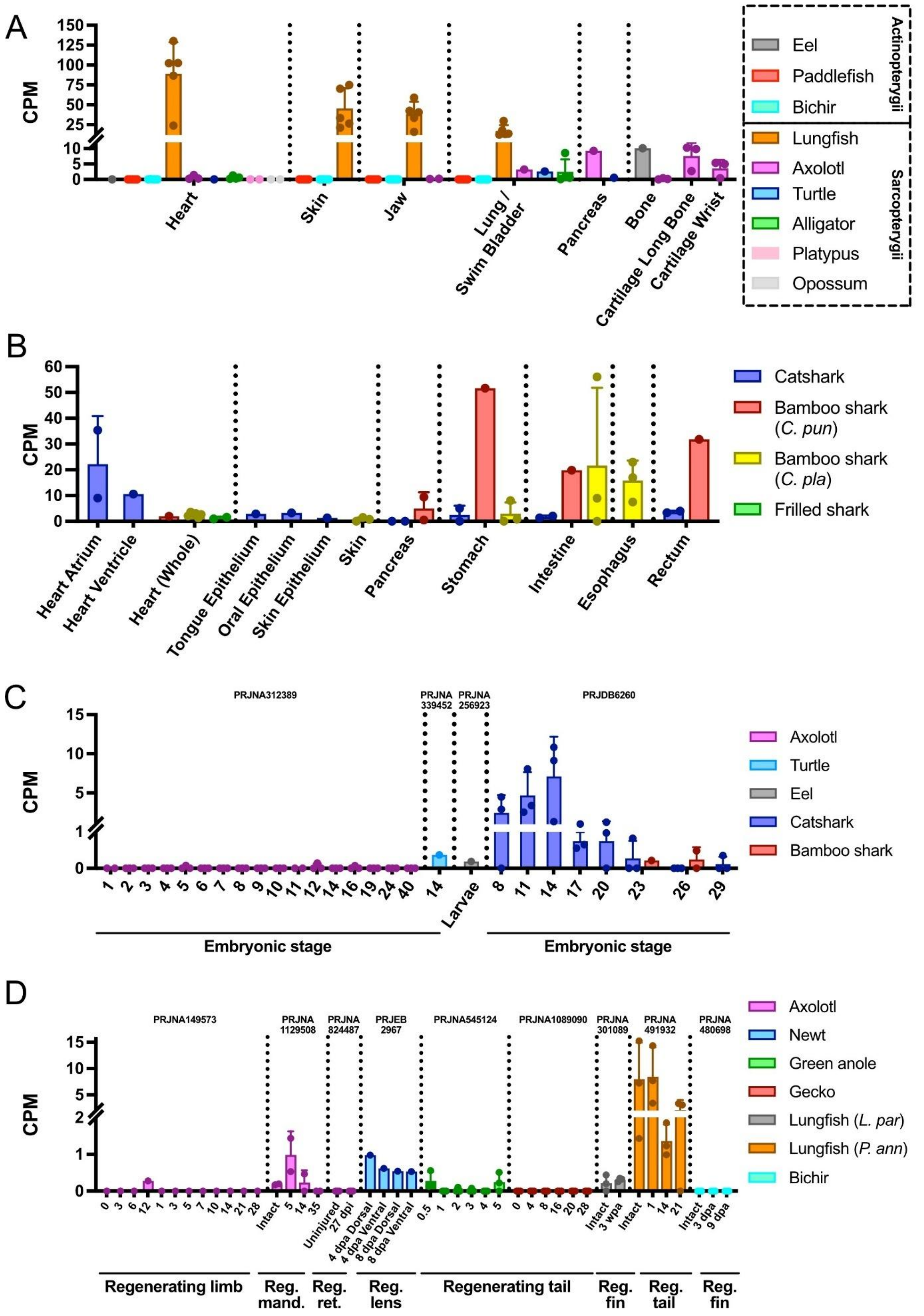

Figure S8. ***Kazald4*** **lacks a conserved expression pattern across jawed vertebrates, related to Figure 3.**

(A) Quantification of *Kazald4* expression in different tissues of multiple sarcopterygian and actinopterygian species. Data is from PRJNA256923, PRJNA599026, PRJNA300706, PRJNA339452, PRJNA556093, PRJNA143627, PRJNA1129508, and PRJNA378982. (B) Quantification of *Kazald4* expression in different tissues of multiple chondrichthyan species. Data is from PRJDB6260, PRJNA1026724, and PRJDB14248. (C) Quantification of *Kazald4* expression in whole embryos during embryonic development of multiple species of jawed vertebrates. (D) Quantification of *Kazald4* expression during regeneration in different tissues of multiple species of bony vertebrates. PRJ IDs indicate the publicly available RNA-Seq datasets which generated the raw data for the listed tissues. Dots represent biological replicates in examined datasets, error bars represent standard deviation when calculable. CPM = Counts Per Million, Reg. = regenerating, mand. = mandible, ret. = retina. Data are represented as Mean ± SD.

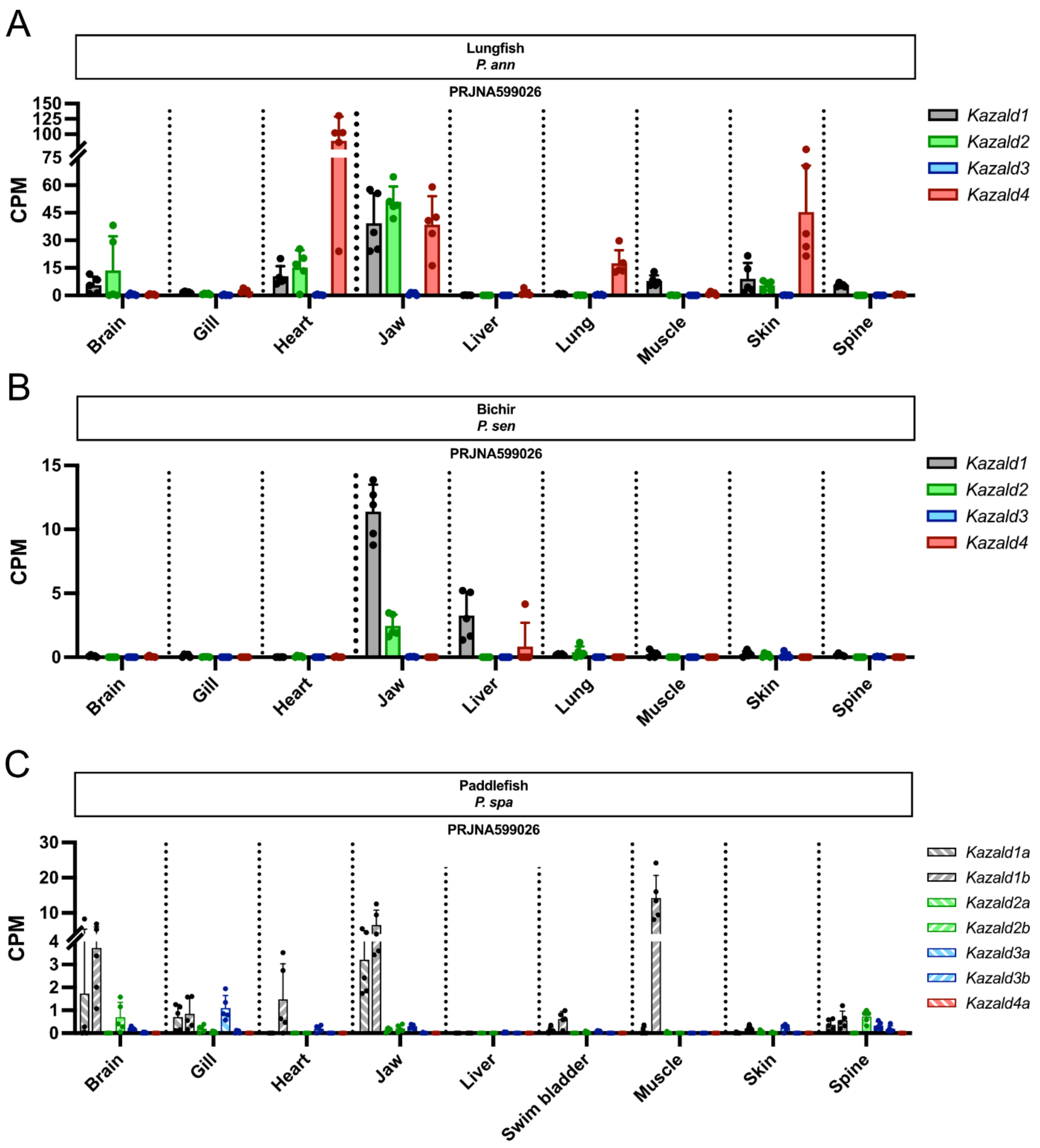

Figure S9. Kazald gene expression in various tissues of non-tetrapod/teleost fish, related to Figures 4 and 5.
(A) Quantification of the expression of all Kazald genes in various tissues of the lungfish. (B) Quantification of the expression of all Kazald genes in various tissues of the bichir. (C) Quantification of the expression of all Kazald genes in various tissues of the paddlefish. PRJ IDs indicate the publicly available RNA-Seq datasets which generated the raw data for the listed tissues. Dots represent biological replicates in examined datasets, error bars represent standard deviation when calculable. CPM = Counts Per Million. Data are represented as Mean ± SD.

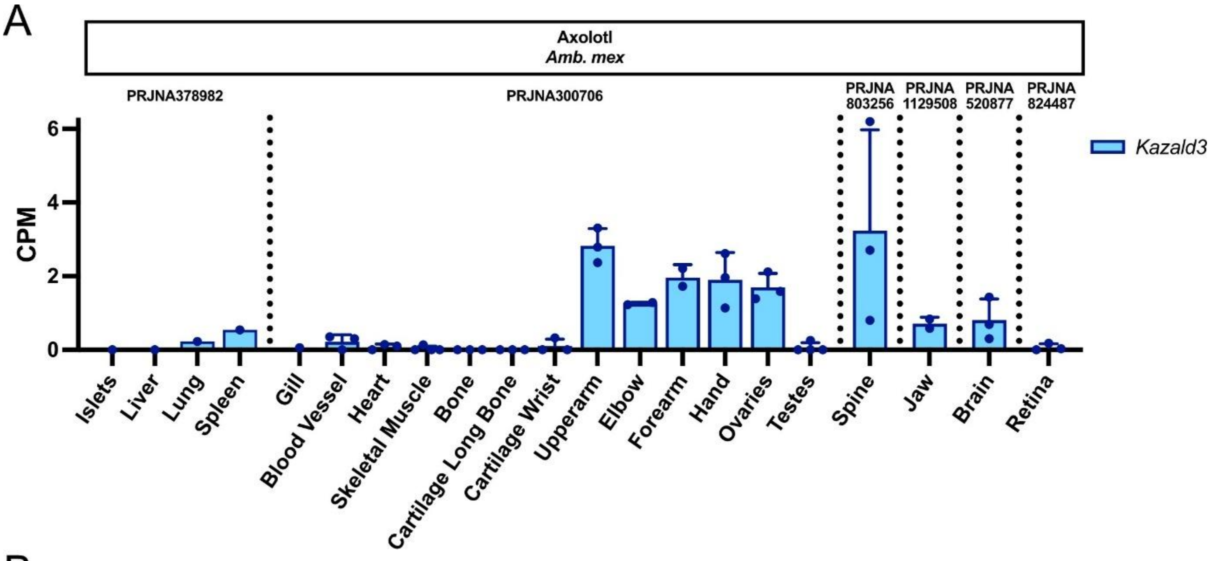

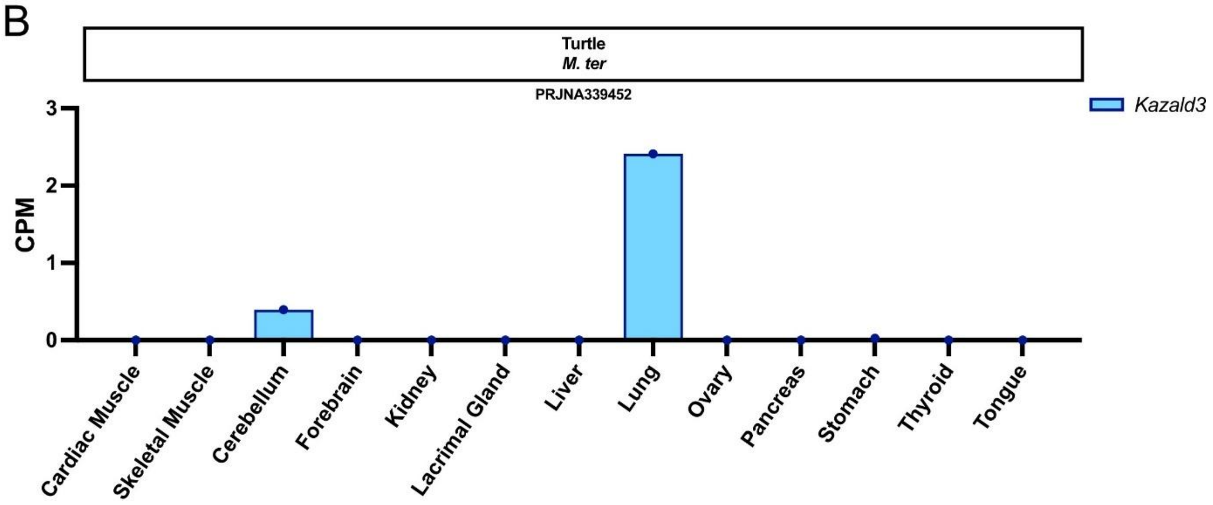

Figure S10. *Kazald3* has no to very low levels of expression in the tissues of adult tetrapods, related to Figure 5.

(A) Quantification of *Kazald3* expression in various tissues of the axolotl. (B) Quantification of *Kazald3* expression in various tissues of the turtle. PRJ IDs indicate the publicly available RNA-Seq datasets which generated the raw data for the listed tissues. Dots represent biological replicates in examined datasets, error bars represent standard deviation when calculable. CPM = Counts Per Million. Data are represented as Mean ± SD.

Table S1. TBLASTN search of the axolotl genome reveals four regions spread across four chromosomes with high similarity to known Kazald genes, related to Table 1.

	**Mouse *Kazald1***						
Axolotl Chromosome	Exon	Start	End	Direction	Identities	Positives	E Value
Chr8	Exon 1	1,479,087,933	1,479,088,340	–	91/137 (66%)	108/137 (79%)	7.00E-54
	Exon 2	1,479,013,148	1,479,013,306	–	48/53 (91%)	50/53 (94%)	4.00E-26
	Exon 3	1,478,969,760	1,478,969,906	–	35/49 (71%)	43/49 (88%)	7.00E-19
Chr3q	Exon 1	561,620,191	561,620,586	+	74/135 (55%)	94/135 (70%)	2.00E-41
	Exon 2	561,884,219	561,884,377	+	31/53 (58%)	36/53 (68%)	2.00E-13
	Exon 3	561,933,219	561,933,356	+	27/46 (59%)	31/46 (67%)	5.00E-12
Chr10	Exon 1	222,256,888	222,257,250	–	63/123 (51%)	81/123 (66%)	3.00E-26
	Exon 2	222,122,948	222,123,106	–	32/53 (60%)	40/53 (75%)	1.00E-15
	Exon 3	222,098,653	222,098,790	–	23/46 (50%)	31/46 (67%)	3.00E-09
Chr6q	Exon 1	1,617,595,903	1,617,596,295	–	63/132 (48%)	82/132 (62%)	2.00E-27
	Exon 2	1,617,577,351	1,617,577,509	–	25/53 (47%)	37/53 (70%)	2.00E-10
	Exon 3	1,617,560,918	1,617,561,055	–	24/46 (52%)	32/46 (70%)	8.00E-11
	**Zebrafish *kazald2***						
Axolotl Chromosome	Exon	Start	End	Direction	Identities	Positives	E Value
Chr6q	Exon 1	1,617,595,903	1,617,596,289	–	68/136 (50%)	80/136 (59%)	9.00E-28
	Exon 2	1,617,577,351	1,617,577,509	–	25/53 (47%)	37/53 (70%)	1.00E-10
	Exon 3	1,617,560,921	1,617,561,058	–	27/46 (59%)	36/46 (78%)	4.00E-13
Chr3q	Exon 1	561,620,212	561,620,583	+	57/132 (43%)	77/132 (58%)	8.00E-23
	Exon 2	561,884,219	561,884,377	+	23/53 (43%)	35/53 (66%)	1.00E-10
	Exon 3	561,933,216	561,933,353	+	27/46 (59%)	35/46 (76%)	1.00E-13
Chr8	Exon 1	1,479,087,933	1,479,088,313	–	55/134 (41%)	77/134 (57%)	3.00E-21
	Exon 2	1,479,013,148	1,479,013,306	–	25/53 (47%)	32/53 (60%)	3.00E-09
	Exon 3	1,478,969,769	1,478,969,903	–	26/45 (58%)	34/45 (76%)	2.00E-11
Chr10	Exon 1	222,256,888	222,257,241	–	60/123 (49%)	73/123 (59%)	2.00E-19
	Exon 2	222,122,948	222,123,106	–	22/53 (42%)	31/53 (58%)	3.00E-09
	Exon 3	222,098,656	222,098,793	–	25/46 (54%)	34/46 (74%)	6.00E-11

Scores are given for the individual exons of the Kazald genes. Identities are number of identical amino acids. Positives are aligned amino acids that are either identical or have similar chemical properties. E Value is the number of expected hits of similar quality that could be found just by chance. Exon 4 is not included due to failure to find a match in the genome.

Table S2. Most jawed vertebrates possess multiple Kazald genes, related to Figure 1.

Common Name	Scientific Name	Total Number of Kazald Genes	Number of Manually Found or Edited Genes
Sponge	*E. muelleri*	–	–
Sponge	*O. lobularis*	–	–
Placozoa	*T. adhaerens*	–	–
Hydra	*H. vulgaris*	–	–
Staghorn Coral	*A. muricata*	–	–
Starlet Sea Anemone	*N. vectensis*	–	–
Flame Jellyfish	*R. esculentum*	–	–
Fruit Fly	*D. melanogaster*	–	–
Nematode	*C. elegans*	–	–
Planaria	*S. mediterranea*	–	–
Planaria	*D. japonica*	–	–
Milk-White Planarian	*D. lacteum*	1*,**	–
Atlantic Horseshoe Crab	***L. pol**yphemus*	1*** (3)	–
Cactus Worm	***P. cau**datus*	1	–
Portuguese Oyster	***C. ang**ulata*	1	–
Leech	***H. rob**usta*	1	–
Acorn Worm	***S. kow**alevskii*	1	–
Crown-of-Thorns Starfish	***A. pla**nci*	1	–
Florida Lancelet	***B. flo**ridae*	1	–
Inshore Hagfish	***E. bur**geri*	1	–
Sea Lamprey	***P. mar**inus*	1	–
Elephant Shark	***C. mil**ii*	2	–
Great White Shark	***C. car**charias*	2	–
Thorny Skate	***A. rad**iata*	2	–
Gray Bichir	***P. sen**egalus*	4	–
Sterlet	***A. rut**henus*	7	2
American Paddlefish	***P. spa**thula*	7	2
Spotted Gar	***L. ocu**latus*	3	–

European Eel	*__A. ang__uilla*	4	1
Atlantic Tarpon	*__M. atl__anticus*	4	3
Asian Bonytongue	*__S. for__mosus*	2	–
Atlantic Herring	*__C. har__engus*	3	1
Allis Shad	*__A. alo__sa*	3	1
Mexican Tetra	*__Ast. mex__icanus*	3	1
Red-Bellied Piranha	*__P. nat__tereri*	3	–
Zebrafish	*__D. rer__io*	2	–
Northern Pike	*__E. luc__ius*	2	1
Chinook Salmon	*__O. tsh__awytscha*	2	–
Atlantic Cod	*__G. mor__hua*	2	–
European Seabass	*__D. lab__rax*	2	–
Torafugu Pufferfish	*__T. rub__ripes*	1	–
Medaka	*__O. lat__ipes*	1	–
Coelacanth	*__L. cha__lumnae*	3	–
West African Lungfish	*__P. ann__ectens*	4	–
Axolotl	*__Amb. mex__icanum*	4	–
Iberian Ribbed Newt	*__P. wal__tl*	3**** (4)	–
Eastern Newt	*__N. vir__idescens*	3**	–
Ramos' Mushroom-Tongue Salamander	*__B. ram__osi*	4**	–
Two-Lined Caecilian	*__R. biv__ittatum*	3	2
Gaboon Caecilian	*__G. ser__aphini*	2	1
Tiny Cayenne Caecilian	*__M. uni__color*	2	–
European Fire-Bellied Toad	*__B. bom__bina*	3	–
Western Clawed Frog	*__X. tro__picalis*	1	–
Common Toad	*__B. buf__o*	2	–
Common Frog	*__R. tem__poraria*	2	–
Painted Turtle	*__C. pic__ta*	3	–
Goode's Thornscrub Tortoise	*__G. evg__oodei*	3	–

Green Sea Turtle	***C. myd***_as_	2	–
Yangtze Giant Softshell Turtle	***R. swi***_nhoei_	2	2
Red-Bellied Short-Necked Turtle	***E. subg***_lobosa_	2	2
Gharial	***G. gan***_geticus_	2	–
Chinese Alligator	***A. sin***_ensis_	2	–
Emu	***Dro. nov***_aehollandiae_	2	–
Chicken	***G. gal***_lus_	2	–
Zebra Finch	***T. gut***_tata_	2	–
Tuatara	***S. pun***_ctatus_	3	2
Leopard Gecko	***E. mac***_ularius_	2	–
Sand Lizard	***L. agi***_lis_	2	–
Eastern Fence Lizard	***S. und***_ulatus_	2	–
Western Terrestrial Garter Snake	***T. ele***_gans_	1	–
Platypus	***O. ana***_tinus_	2	–
Short-Beaked Echidna	***T. acu***_leatus_	2	1
Gray Short-Tailed Opossum	***M. dom***_estica_	2	1
Common Brushtail Possum	***T. vul***_pecula_	2	–
Tasmanian Devil	***S. har***_risii_	2	1
Indian Elephant	***E. max. ind***_icus_	1	–
Nine-Banded Armadillo	***Das. nov***_emcinctus_	1	–
Horse	***E. cab***_allus_	1	–
Large Flying Fox	***P. vam***_pyrus_	1	–
Dog	***C. lup. fam***_iliaris_	1	–
Mouse	***M. mus***_culus_	1	–
Human	***H. sap***_iens_	1	–

Putative Kazald genes from the species which possessed them were used for the creation of the large-scale phylogenetic tree (Figure 1). Bolded letters in the column “Scientific Name” are used as the IDs of species in the tree. The column “Number of Manually Found or Edited Genes” refers to genes either not in the transcriptome, or manually edited based on the conserved Kazald gene genomic profile that we identified (see Materials and Methods). Use of – indicates absence. * = not used in the large-scale phylogenetic tree. ** = only transcriptomes are available, thus the total number of Kazald genes may

be greater. *** = 3 potential Kazald genes found broken up across scaffolds, thus only the 1 fully intact gene used in the phylogenetic tree. **** = 4 Kazald genes found in the genome, but only the 3 genes in the transcriptome used in the phylogenetic tree.

Supplemental References

1. L. Wu, X. Xia, X. Pan, A novel score for highly accurate and efficient prediction of native protein structures. [Preprint] (2020). Available at: https://www.biorxiv.org/content/10.1101/2020.04.23.056945v1 [Accessed 20 June 2025].

2. L. Xu, S. Y. Wa Sin, P. Grayson, S. V. Edwards, T. B. Sackton, Evolutionary Dynamics of Sex Chromosomes of Paleognathous Birds. *Genome Biol. Evol.* **11**, 2376–2390 (2019).

3. G. Gedman, *et al.*, As above, so below: Whole transcriptome profiling demonstrates strong molecular similarities between avian dorsal and ventral pallial subdivisions. *J. Comp. Neurol.* **529**, 3222–3246 (2021).

4. D. Brawand, *et al.*, The evolution of gene expression levels in mammalian organs. *Nature* **478**, 343–348 (2011).

5. J. A. St John, *et al.*, Sequencing three crocodilian genomes to illuminate the evolution of archosaurs and amniotes. *Genome Biol.* **13**, 415 (2012).

6. S. Yang, *et al.*, Genomic investigation of the Chinese alligator reveals wild-extinct genetic diversity and genomic consequences of their continuous decline. *Mol. Ecol. Resour.* **23**, 294–311 (2023).

7. H. Jiang, *et al.*, Chromosome-level genome of diamondback terrapin provides insight into the genetic basis of salinity adaptation. *Integr. Zool.* **n/a**.

8. S. Schloissnig, *et al.*, The giant axolotl genome uncovers the evolution, scaling, and transcriptional control of complex gene loci. *Proc. Natl. Acad. Sci.* **118**, e2017176118 (2021).

9. S. Nowoshilow, *et al.*, The axolotl genome and the evolution of key tissue formation regulators. *Nature* **554**, 50–55 (2018).

10. A. S. Yandulskaya, M. N. Miller, R. Ansaripour, R. L. Carrier, J. R. Monaghan, Regenerating axolotl retinas regrow diverse cell types with modulation by Notch signaling and reconnect to the brain. [Preprint] (2022). Available at: https://www.biorxiv.org/content/10.1101/2022.04.28.489898v1 [Accessed 4 August 2025].

11. X. Bi, *et al.*, Tracing the genetic footprints of vertebrate landing in non-teleost ray-finned fishes. *Cell* **184**, 1377-1391.e14 (2021).

12. J. Pasquier, *et al.*, Gene evolution and gene expression after whole genome duplication in fish: the PhyloFish database. *BMC Genomics* **17**, 368 (2016).

13. Y. Hara, *et al.*, Shark genomes provide insights into elasmobranch evolution and the origin of vertebrates. *Nat. Ecol. Evol.* **2**, 1761–1771 (2018).

14. O. Nishimura, *et al.*, Squalomix: shark and ray genome analysis consortium and its data sharing platform. [Preprint] (2025). Available at: https://f1000research.com/articles/11-1077 [Accessed 4 August 2025].

15. P. He, *et al.*, The changing mouse embryo transcriptome at whole tissue and single-cell resolution. *Nature* **583**, 760–767 (2020).

16. I. Dunham, *et al.*, An integrated encyclopedia of DNA elements in the human genome. *Nature* **489**, 57–74 (2012).

17. Y. Luo, *et al.*, New developments on the Encyclopedia of DNA Elements (ENCODE) data portal. *Nucleic Acids Res.* **48**, D882–D889 (2020).

18. B. C. Hitz, *et al.*, The ENCODE Uniform Analysis Pipelines. [Preprint] (2023). Available at: https://www.biorxiv.org/content/10.1101/2023.04.04.535623v1 [Accessed 4 August 2025].

19. P. Jiang, C. S. Chamberlain, R. Vanderby, J. A. Thomson, R. Stewart, TimeMeter assesses temporal gene expression similarity and identifies differentially progressing genes. *Nucleic Acids Res.* **48**, e51 (2020).

20. F. Qu, I. C. Palte, P. M. Gontarz, B. Zhang, F. Guilak, Transcriptomic analysis of bone and fibrous tissue morphogenesis during digit tip regeneration in the adult mouse. *FASEB J.* **34**, 9740–9754 (2020).

21. P. Gomez-Picos, K. Ovens, B. F. Eames, Limb Mesoderm and Head Ectomesenchyme Both Express a Core Transcriptional Program During Chondrocyte Differentiation. *Front. Cell Dev. Biol.* **10** (2022).

22. P. Jiang, *et al.*, Analysis of embryonic development in the unsequenced axolotl: Waves of transcriptomic upheaval and stability. *Dev. Biol.* **426**, 143–154 (2017).

23. R. Stewart, *et al.*, Comparative RNA-seq Analysis in the Unsequenced Axolotl: The Oncogene Burst Highlights Early Gene Expression in the Blastema. *PLOS Comput. Biol.* **9**, e1002936 (2013).

24. J. Kramer, *et al.*, Axolotl mandible regeneration occurs through mechanical gap closure and a shared regenerative program with the limb. *Dis. Model. Mech.* **17**, dmm050743 (2024).

25. D. M. Bryant, *et al.*, A Tissue-Mapped Axolotl De Novo Transcriptome Enables Identification of Limb Regeneration Factors. *Cell Rep.* **18**, 762–776 (2017).

26. Y. Zhang, *et al.*, The White-Spotted Bamboo Shark Genome Reveals Chromosome Rearrangements and Fast-Evolving Immune Genes of Cartilaginous Fish. *iScience* **23**, 101754 (2020).

27. Y. Li, *et al.*, Origin and stepwise evolution of vertebrate lungs. *Nat. Ecol. Evol.* **9**, 672–691 (2025).

28. S. Darnet, *et al.*, Deep evolutionary origin of limb and fin regeneration. *Proc. Natl. Acad. Sci.* **116**, 15106–15115 (2019).

29. J.-L. Hou, *et al.*, Integration of Metabolomics and Transcriptomics to Reveal the Metabolic Characteristics of Exercise-Improved Bone Mass. *Nutrients* **15**, 1694 (2023).

30. R. F. Bloomquist, *et al.*, Developmental plasticity of epithelial stem cells in tooth and taste bud renewal. *Proc. Natl. Acad. Sci.* **116**, 17858–17866 (2019).

31. K. M. Verissimo, *et al.*, Salamander-like tail regeneration in the West African lungfish. *Proc. R. Soc. B Biol. Sci.* **287**, 20192939 (2020).

32. S. E. Walker, K. Z. Sabin, M. D. Gearhart, K. Yamamoto, K. Echeverri, Regulation of stem cell identity by miR-200a during spinal cord regeneration. *Development* **149**, dev200033 (2022).

33. K. Sousounis, *et al.*, Transcriptome Analysis of Newt Lens Regeneration Reveals Distinct Gradients in Gene Expression Patterns. *PLOS ONE* **8**, e61445 (2013).

34. M. Looso, *et al.*, A de novo assembly of the newt transcriptome combined with proteomic validation identifies new protein families expressed during tissue regeneration. *Genome Biol.* **14**, R16 (2013).

35. P. E. Herman, *et al.*, Highly conserved molecular pathways, including Wnt signaling, promote functional recovery from spinal cord injury in lampreys. *Sci. Rep.* **8**, 742 (2018).

36. Y. Liang, *et al.*, BMP signaling is required for amphioxus tail regeneration. *Development* **146**, dev166017 (2019).

37. J. Kang, *et al.*, Modulation of tissue repair by regeneration enhancer elements. *Nature* **532**, 201–206 (2016).

38. C. Xu, E. D. Hutchins, M. A. Tokuyama, J. Wilson-Rawls, K. Kusumi, Transcriptional analysis of scar-free wound healing during early stages of tail regeneration in the green anole lizard, *Anolis carolinensis*. *J. Immunol. Regen. Med.* **7**, 100025 (2020).

39. L. Nurhidayat, *et al.*, Tokay gecko tail regeneration involves temporally collinear expression of HOXC genes and early expression of satellite cell markers. *BMC Biol.* **23**, 6 (2025).

40. J. Zhong, *et al.*, Multi-species atlas resolves an axolotl limb development and regeneration paradox. *Nat. Commun.* **14**, 6346 (2023).

41. S.-Y. Liu, *et al.*, Reactivating head regrowth in a regeneration-deficient planarian species. *Nature* **500**, 81–84 (2013).

42. J. Pascual-Anaya, *et al.*, Hagfish and lamprey Hox genes reveal conservation of temporal colinearity in vertebrates. *Nat. Ecol. Evol.* **2**, 859–866 (2018).

43. S. A. Bryant, J. R. Herdy, C. T. Amemiya, J. J. Smith, Characterization of Somatically-Eliminated Genes During Development of the Sea Lamprey (Petromyzon marinus). *Mol. Biol. Evol.* **33**, 2337–2344 (2016).

44. A. Angeloni, *et al.*, Extensive DNA methylome rearrangement during early lamprey embryogenesis. *Nat. Commun.* **15**, 1977 (2024).

45. F. Marlétaz, *et al.*, Amphioxus functional genomics and the origins of vertebrate gene regulation. *Nature* **564**, 64–70 (2018).

46. D. V. Dylus, *et al.*, Large-scale gene expression study in the ophiuroid Amphiura filiformis provides insights into evolution of gene regulatory networks. *EvoDevo* **7**, 2 (2016).

47. T. Gildor, G. A. Cary, M. Lalzar, V. F. Hinman, S. Ben-Tabou de-Leon, Developmental transcriptomes of the sea star, Patiria miniata, illuminate how gene expression changes with evolutionary distance. *Sci. Rep.* **9**, 16201 (2019).

48. M. R. Hall, *et al.*, The crown-of-thorns starfish genome as a guide for biocontrol of this coral reef pest. *Nature* **544**, 231–234 (2017).

49. F. Marlétaz, *et al.*, The hagfish genome and the evolution of vertebrates. *Nature* **627**, 811–820 (2024).

50. S. D. Vianello, *et al.*, Deconstructing the common anteroposterior organisation of adult bilaterian guts. [Preprint] (2025). Available at: https://www.biorxiv.org/content/10.1101/2025.07.02.662275v1 [Accessed 14 January 2026].

51. S. Matsubara, T. Osugi, A. Shiraishi, A. Wada, H. Satake, Comparative analysis of transcriptomic profiles among ascidians, zebrafish, and mice: Insights from tissue-specific gene expression. *PLOS ONE* **16**, e0254308 (2021).

52. E. K. Lowe, A. L. Garm, E. Ullrich-Lüter, C. Cuomo, M. I. Arnone, The crowns have eyes: multiple opsins found in the eyes of the crown-of-thorns starfish Acanthaster planci. *BMC Evol. Biol.* **18**, 168 (2018).

53. H. Yuasa, *et al.*, Elucidation of the speciation history of three sister species of crown-of-thorns starfish (Acanthaster spp.) based on genomic analysis. *DNA Res.* **28**, dsab012 (2021).
